# The effect of ionic strength on PETase enzymes: An experimental and computational study

**DOI:** 10.1002/pro.70386

**Published:** 2025-12-27

**Authors:** Alessandro Berselli, Alan Carletti, Maria Cristina Menziani, Shapla Bhattacharya, Rossella Castagna, Emilio Parisini, Giulia di Rocco, Francesco Muniz‐Miranda

**Affiliations:** ^1^ Department of Chemical and Geological Sciences (DSCG) University of Modena and Reggio Emilia (UNIMORE) Modena Italy; ^2^ Department of Life Sciences (DSV) University of Modena and Reggio Emilia (UNIMORE) Modena Italy; ^3^ Department of Biotechnology, Latvian Institute of Organic Synthesis Riga Latvia; ^4^ Faculty of Natural Sciences and Technology, Riga Technical University Riga Latvia; ^5^ Department of Chemistry, Materials and Chemical Engineering “G. Natta”, Politecnico di Milano Milan Italy; ^6^ Department of Chemistry “G. Ciamician” University of Bologna Bologna Italy

**Keywords:** Ideonella Sakaiensis 201‐f6, molecular dynamics simulations, PETase, salt tolerance, Streptomyces sp. SM14

## Abstract

Over recent decades, various enzymes capable of breaking down polyethylene terephthalate (PET) have emerged as sustainable tools for plastic waste management. Among them, IsPETase from *Ideonella sakaiensis* 201‐f6 stands out for its high catalytic activity at low temperatures. However, the discovery of the PETase‐like enzyme from the marine sponge *Streptomyces* sp. SM14 (PETaseSM14) has introduced a new class of biocatalysts active at high‐salt concentrations, whose structural and catalytic properties remain poorly understood. This study explores the structural and catalytic behavior of both IsPETase and PETaseSM14 under varying ionic strength (from 150 to 900 mM of NaCl concentration) using all‐atom molecular dynamics simulations and in vitro assays. Results reveal that the flexible, enlarged binding site of IsPETase improves substrate accommodation but also causes catalytic residue displacement and rapid deactivation, particularly under high‐salt conditions. In contrast, PETaseSM14 has a smaller, more rigid binding pocket that undergoes moderate widening upon salt concentration increasing, thus promoting water and substrate recruitment. Additionally, active forms of both enzymes bind PET chains in conformations similar to those found in amorphous PET. These findings offer key structural insights that can inform future enzyme engineering efforts for effective PET degradation tailored to diverse environmental conditions.

**Abbreviations**
AFM: atomic force microscopy
BHET: bis(hydroxyethyl) terephthalate
CG: coarse‐grained
CGenFF: CHARMM general force field
DFT: density functional theory
HPLC: high performance liquid chromatography
MD: molecular dynamics
MHET: mono(hydroxyethyl) terephthalate
PAZy: plastic‐active enzymes database
PBCs: periodic boundary conditions
PET: polyethylene terephthalate
QM/MM: quantum mechanics/molecular mechanics
RMSD: root‐mean‐square deviation
RMSF: root‐mean‐square fluctuation
SASA: solvent accessible surface area
TPA: terephthalic acid
WT: wild type

## 1 INTRODUCTION

The abuse and improper waste management of plastic materials has become a pressing issue for human health. Due to their persistency in nature, plastics accumulated in the environment are fragmented into smaller pieces, originating microplastics that migrate via rivers and oceans, forming accumulating zones named “garbage patches” (Lee et al., 2022). As a consequence of the widespread diffusion of these exogenous materials in the habitats, many organisms adapted their metabolism to use plastic compounds as a new source of carbon, leading to the development of several plastic‐degrading enzymes (Ruginescu & Purcarea, 2024).

The first reports of microbial strains capable of degrading aliphatic synthetic polyesters date back to the 1970s (Potts et al., 1973; Tokiwa & Suzuki, 1977), but the role of enzymes in plastic depolymerization was recognized about 30 years later, with the documentation of the biocatalytic hydrolysis of polyethylene terephthalate (PET) (Müller et al., 2005). Since that discovery, the number of PET‐active enzymes isolated and characterized increased considerably, offering a great opportunity for humans to exploit the naturally evolved biotechnological systems to face the environmental challenge of plastic pollution (Samak et al., 2020; Wei & Zimmermann, 2017; Zimmermann & Billig, 2011).

Today, the plastic‐active enzymes database (PAZy) (Buchholz et al., 2022) includes 311 distinct wild‐type (WT) PET hydrolytic enzymes biochemically characterized. Most of them exhibit peak activity under thermophilic conditions, close to the glass transition temperature of PET (~61°C for amorphous dry PET and ~79°C for high‐crystallinity dry PET, reduced by up to 16°C in water; Chen et al., 1998; Groeninckx et al., 1974; Launay et al., 1999), due to the formation of flexible and enzyme‐accessible amorphous domains (Alves et al., 2002). The isolation of the enzyme PETase from the bacterial strain *Ideonella Sakaiensis* 201‐f6 (referred to as IsPETase hereafter) (Yoshida et al., 2016), in 2016, represented a turning point in this field. By converting PET mainly into mono‐ and bis‐hydroxyethyl terephthalate (MHET, BHET) and terephthalic acid (TPA), this enzyme outperformed other homologous cutinases (Müller et al., 2005; Silva et al., 2005; Sulaiman et al., 2012; Yoshida et al., 2016) in terms of catalytic activity at low temperatures thanks to the enhanced flexibility of its binding site, which facilitates the substrate recruitment even for relatively rigid PET chains (Berselli et al., 2021; Fecker et al., 2018). As a drawback of the higher binding site's plasticity, the catalytic efficiency of IsPETase drops at higher temperatures, hindering industrial application (de Castro et al., 2017; Kawai et al., 2024). For this reason, in recent years, huge efforts have been made to engineer mutants of IsPETase featuring higher catalytic activity and thermostability (Arnal et al., 2023; Bell et al., 2022; Cui et al., 2021; Cui et al., 2024; Joo et al., 2018; Lu et al., 2022; Son et al., 2019; Sun et al., 2021). While IsPETase adapted to work at relatively low temperatures (~37°C), the structure of other PETase‐like enzymes optimized towards the conditions of their native habitat. A remarkable example is the marine‐sponge derived *Streptomyces* sp. SM14 PETase (referred to as PETaseSM14 hereafter) (Almeida et al., 2019; Carr et al., 2023). Although belonging to the same family as IsPETase, this enzyme exhibits distinctive structural characteristics that evolved to adapt to the high‐salt conditions of the marine environment. Indeed, in a previous study performed by some of us (Carletti et al., 2025), it was shown that PETaseSM14 exerts catalytic activity enhanced in the presence of high concentrations of NaCl, peaking at 900 mM. This characteristic is contrary to that observed for IsPETase, which degrades PET under low salt conditions but quickly deactivates with the increasing ionic strength.

**FIGURE 1:**
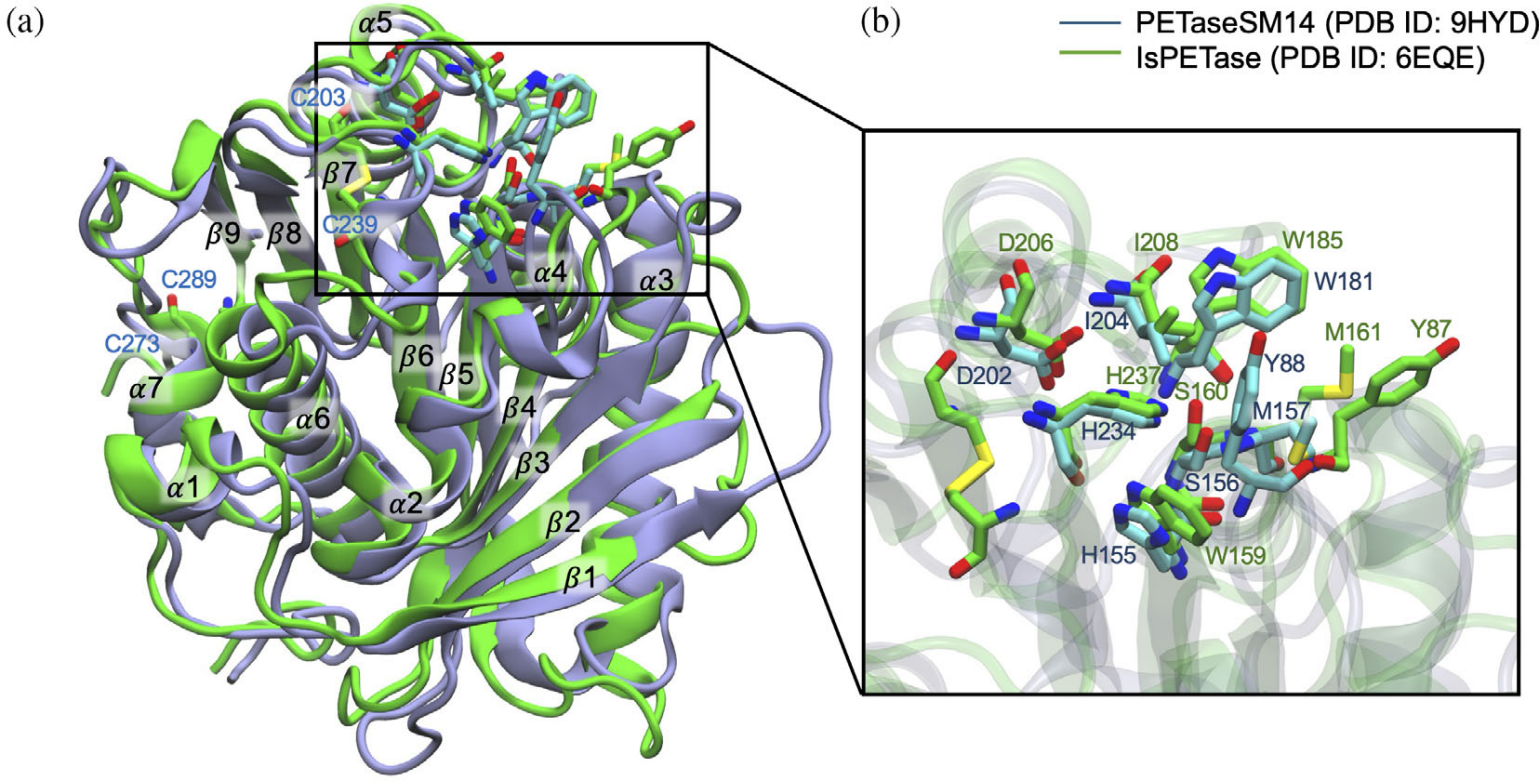
Three‐dimensional structure of PETaseSM14 and IsPETase. (a) Structural superposition of the crystallographic structure of PETaseSM14 (blue structure, PDB ID: 9HYD) (Carletti et al., 2025) and IsPETase (green structure, PDB ID: 6EQE) (Austin et al., 2018). (b) Close‐up view of the enzymes' binding sites.

From a structural point of view, IsPETase and PETaseSM14 are serine hydrolases, characterized by a conserved Gly‐x1‐Ser‐x2‐Gly motif and a catalytic triad composed of serine (S155/S160 in PETaseSM14 and IsPETase, respectively), histidine (H234/H237 in PETaseSM14 and IsPETase, respectively) and aspartate (D202/D206 in PETaseSM14 and IsPETase, respectively) that exert the PET hydrolysis (Almeida et al., 2019; Austin et al., 2018; Berselli et al., 2021; Fecker et al., 2018; Joo et al., 2018). Both enzymes consist of a single domain, resembling the typical folding of an α/β hydrolase (Ollis et al., 1992), characterized by a central nine‐stranded twisted β‐strand, surrounded by seven α‐helices (Figure 1a). The binding site of the two proteins include the catalytic triad, a methionine (M157/M161) and a tyrosine (Y88/Y87), whose backbone form the *oxyanion hole* that stabilize the intermediate states during the reaction (Berselli et al., 2025; Burgin et al., 2024; Jerves et al., 2021). Moreover, a tryptophane (W181/W185) and an isoleucine (I204/I208), together with the tyrosine side chain, constitute a superficial hydrophobic scaffold that anchor the PET chains for binding (Figure 1b) (Berselli et al., 2024; Joo et al., 2018).

However, relevant differences exist between the two enzymes. For instance, the IsPETase W159 is replaced by a histidine (H155) in PETaseSM14 (residue x1 in the Gly‐x1‐Ser‐x2‐Gly motif). Moreover, IsPETase is characterized by (i) an extra turn extension of the α 2‐helix due to a three‐residue insertion (Figure S1), (ii) the inclusion of three 3_10_‐helices, and (iii) the presence of two disulfide bonds (C203‐C239 and C273‐C289) that confer enhanced flexibility to the binding site, helping substrate recognition and binding (Berselli et al., 2021; Fecker et al., 2018).

Although the halophilic nature of PETaseSM14 and the low salt tolerance of IsPETase have been experimentally assessed (Almeida et al., 2019; Carletti et al., 2025; Carr et al., 2023), a precise molecular understanding of how salt concentrations affects their structure and PET binding at the catalytic site is still lacking.

In this study, we expand our previous assessment of the salt‐concentration‐dependent enzymatic activities of PETaseSM14 and IsPETase (originally performed on post‐consumer plastics) (Carletti et al., 2025) by using PET powder as a uniform substrate. We combine experimental assays with all‐atom molecular dynamics (MD) simulations to investigate how different ionic strengths (150 and 900 mM NaCl) influence the structural plasticity of the enzymes' binding sites and their interaction with PET chains modeled as entangled 9‐mer slabs (Sahihi et al., 2024).

Our findings elucidate the mechanistic differences in flexibility, hydration, and active‐site stability that underlie the divergent salt responses of these homologous enzymes, and provide insights for specific targeted modifications to enhance the catalytic performance of PETase‐like enzymes across different habitats.

## 2 RESULTS

### 2.1 PETaseSM14 and IsPETase exhibit different activity profiles in the presence of NaCl

**FIGURE 2:**
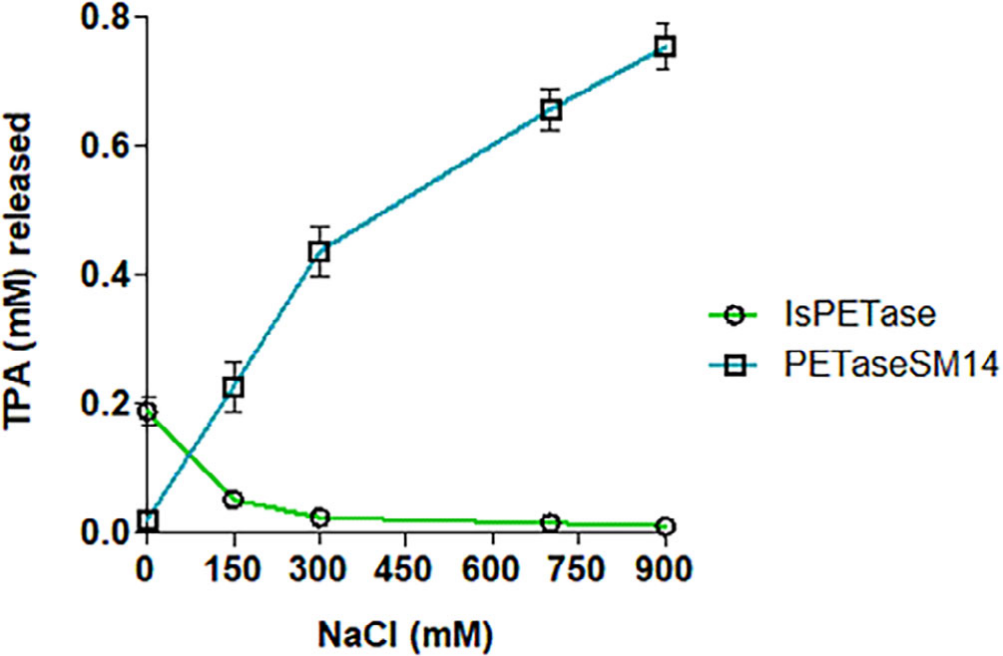
Analysis of the activity in the presence of 0, 150, 300, 700, and 900 mM NaCl on PET powder after 72 h of incubation at 37°C with 400 rpm for (green line) IsPETase, (blue line) PETaseSM14. The protein concentration was 1 μM and the buffer used are: 100 mM Tris–HCl buffer at pH 8.0 for IsPETase and 100 mM Tris–HCl pH 9.0 for PETaseSM14. The data and the associated errors are reported as the mean and standard deviation of the triplicate. When not visible, the error bars fall within the size of the symbols.

The activity of IsPETase and PETaseSM14 was evaluated in previous work using PET film and post‐consumer plastic as substrates (Carletti et al., 2025; Di Rocco et al., 2023). The optimal reaction conditions established in those studies were applied here to assess the enzymatic activity on pure PET powder. PETaseSM14 and IsPETase activities were assessed using 5 mg mL^−1^ of PET powder and allowing the reaction to run for 72 h in the presence of 0, 150, 300, 700, and 900 mM of NaCl. Calibration curves for TPA were generated using standard solutions and the relative standard deviation was evaluated for three consecutive runs, yielding a solid linear relationship (*r* ^2^ = 0.9974 for TPA signal at 1.7 min and *r* ^2^ = 0.9998 for TPA signal at 2.1 min) (Figure S2). The linear regression parameters were then used to quantify the TPA concentration of all samples. The amount of TPA released from the enzymatic activity is reported in Figure 2 as a function of salt concentration, including values at 150 and 900 mM of NaCl, consistent with those used in the simulations reported hereafter. In the presence of NaCl, the two enzymes displayed opposite behaviors: PETaseSM14 exhibited a pronounced increase in TPA production, achieving the highest activity at 900 mM of salts, as remarked in a previous study (Carletti et al., 2025). In contrast, IsPETase activity was drastically reduced in the presence of high salts concentration, showing a decrease of almost 90% in TPA release at 900 mM of NaCl.

Therefore, analytical investigation shows that a greater amount of NaCl exerts a positive impact on PETaseSM14 activity but inhibits IsPETase. However, the structural basis of these effects remains unclear. In the following, we integrate these experimental findings with a detailed in silico analysis to investigate how variations in ionic strength influence the structural properties of the two enzymes and to provide a molecular‐level understanding of their contrasting responses to salt concentration.

### 2.2 PETaseSM14 and IsPETase exhibit different flexibility of the binding domains

Given the different activity observed for the two enzymes at varying ion concentrations, we performed a molecular‐level comparison of their structural properties and their PET‐binding characteristics using MD simulations. To investigate the enzyme‐substrate interactions, each protein was adsorbed onto a PET slab, used as a proxy for a realistic substrate (Sahihi et al., 2024), and solvated at 150 and 900 mM of NaCl concentrations.

The structural stability of these systems was assessed by computing the root‐mean square deviation (RMSD) of the protein backbones over time. The results reported in Figure S3a show that each system reaches a plateau between 1.3 and 1.8 Å after approximately 100 ns of MD simulation, indicative of global stability of the tertiary structures. Despite the similarities in terms of global rearrangements highlighted for the four systems, relevant differences are found in the local displacements of the protein domains, as shown by the root‐mean square fluctuations (RMSF) displayed in Figure S3b. In particular, the regions corresponding to residues from 200 to 210 (labeled as domain “1”) and from 230 to 250 (labeled as domain “2”) include the catalytic residues D202/D206 and H234/H237 in PETaseSM14 and IsPETase, respectively (Figure S3c).

The analysis reveals that amino acid stretches with RMSF values of 1.8 and 2.7 Å for IsPETase (green and red traces for 150 and 900 mM of ion concentration, respectively). In contrast, values below 1 Å are observed for PETaseSM14 at 150 mM (blue trace), with a marginal increase observed at 900 mM (orange trace). This difference is mainly due to the well‐known three‐residue insertion in the α 6–β 8 loop of IsPETase (Figure S1), which confers to the enzyme's binding site a higher flexibility compared to PETaseSM14, and the other homologous serine hydrolases (Austin et al., 2018; Berselli et al., 2021; Berselli et al., 2024; Fecker et al., 2018; Joo et al., 2018). This characteristic is peculiar to IsPETase and it is crucial for the recruitment of the PET substrate, enabling this enzyme to outperform other hydrolases in terms of catalytic efficiency under mild conditions (low temperature and physiological ion concentration) (Fecker et al., 2018; Han et al., 2017; Joo et al., 2018). Additionally, from the superposition of the crystallographic conformations of the binding sites of the two enzymes (Figure 1b, IsPETase in green, PDB ID: 6EQE [Austin et al., 2018], PETaseSM14 in cyan, PDB ID: 9HYD [Carletti et al., 2025]) it can be observed an optimal overlap of the side chains of each amino acid, except for Y87/Y88. Indeed, this residue faces the I204 and W181 side chains in PETaseSM14, whereas it is shifted away from the binding site and exposed to the bulk solvent in IsPETase.

### 2.3 The widening of the IsPETase binding site compromises the stability of the catalytic triad

The structural differences between the binding sites of PETaseSM14 and IsPETase result in distinct degrees of opening and overall size of the cleft, modulating the accessibility of both the solvent and the substrate.

**FIGURE 3:**
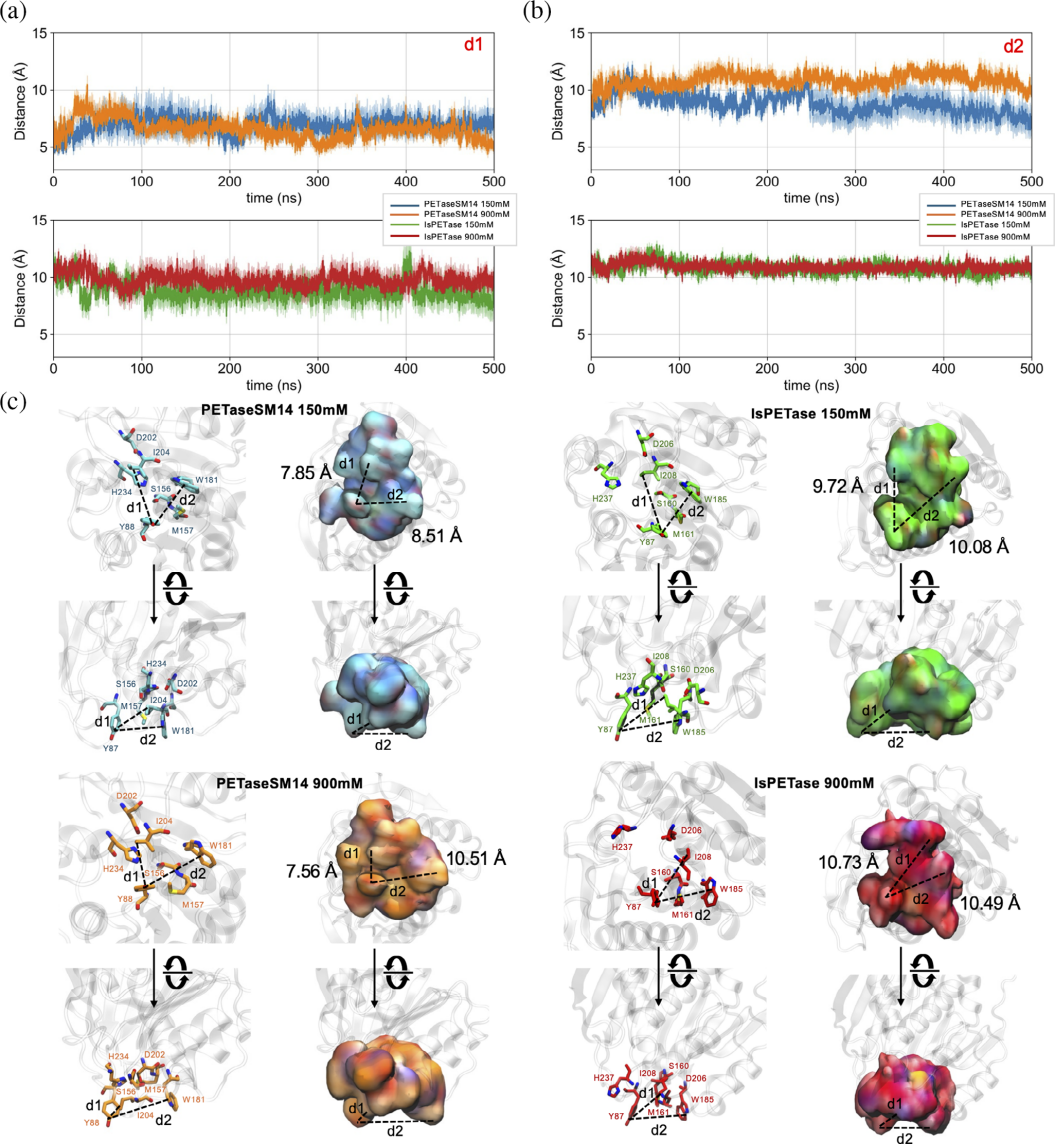
Size of the PETaseSM14 and IsPETase binding sites. The distances between (a) tyrosine and isoleucine side chains (d1) and between (b) the tyrosine and tryptophan side chains (d2) were calculated during the 500 ns‐long MD simulations. The value and the associated error are reported as the average and standard deviation over the three independent replicas performed for each system. (c) Representative snapshots of the PETaseSM14 and IsPETase binding sites at varying NaCl concentrations. The d1 and d2 average values, calculated from MD simulations, are indicated.

To quantitatively assess the extent of cleft opening under different ionic strengths, we measured the inter‐residue distances between the key hydrophobic solvent‐exposed amino acids responsible for the binding and stabilization of the PET chains. The atoms considered in each calculation are listed in Table 1. Specifically, the distances between Y87/Y88 and I208/I204 (d1, Figure 3a), and between Y87/Y88 and W185/W181 (d2, Figure 3b), were used as geometric proxies to estimate the width of the cleft and the surface available for substrate accommodation. In PETaseSM14, d1 remains stable between 6.5 and 7.0 Å at both 150 and 900 mM concentrations (Figure 3a, blue and orange traces, respectively). On the other hand, IsPETase exhibits a considerably larger d1 value of approximately 9 Å, on average, at 150 mM, which increases to around 10.7 Å at 900 mM (Figure 3a, green and red traces, respectively). Regarding d2, PETaseSM14 shows an increase from ~8.5 Å at 150 mM to ~10.5 Å at 900 mM, whereas IsPETase maintains a constant value around 10–10.5 Å across both conditions. These results suggest that the inherently narrower and more rigid cleft of PETaseSM14 undergoes moderate widening at elevated ionic strength, with d2 increasing by 2 Å on average. Conversely, the cleft of IsPETase is already wide at 150 mM and exhibits only a slight expansion at 900 mM.

**TABLE 1:** Atoms used in the calculation of inter‐residue distances.

Distance	System	Residue1 (Atom1)	Residue2 (Atom2)
d1	PETaseSM14	Y88 (OH)	I204 (CD1)
d1	IsPETase	Y87 (OH)	I208 (CD1)
d2	PETaseSM14	Y88 (OH)	W181 (CG)
d2	IsPETase	Y87 (OH)	W185 (CG)
d3	PETaseSM14	S156 (OG)	H234 (ND1)
d3	IsPETase	S160 (OG)	H237 (ND1)
d4	PETaseSM14	H155 (ND1)	N238 (CG)
d4	IsPETase	W159 (CG)	N241 (CG)

*Note*: The atoms considered in the calculation of each distance (d1–d4) for the two enzymes are defined according to the CHARMM nomenclature.

This structural variability has direct implications for substrate accessibility and the stability of the catalytic site. While a more open cleft in IsPETase may facilitate rapid substrate recruitment, excessive widening, particularly under high salt conditions, could destabilize the local interaction network that supports the catalytic triad. Disruption of this architecture may impair the enzymatic activity, as observed experimentally under high ionic strength (Carletti et al., 2025).

### 2.4 Histidine 155 helps stabilizing the catalytic triad in PETaseSM14

**FIGURE 4:**
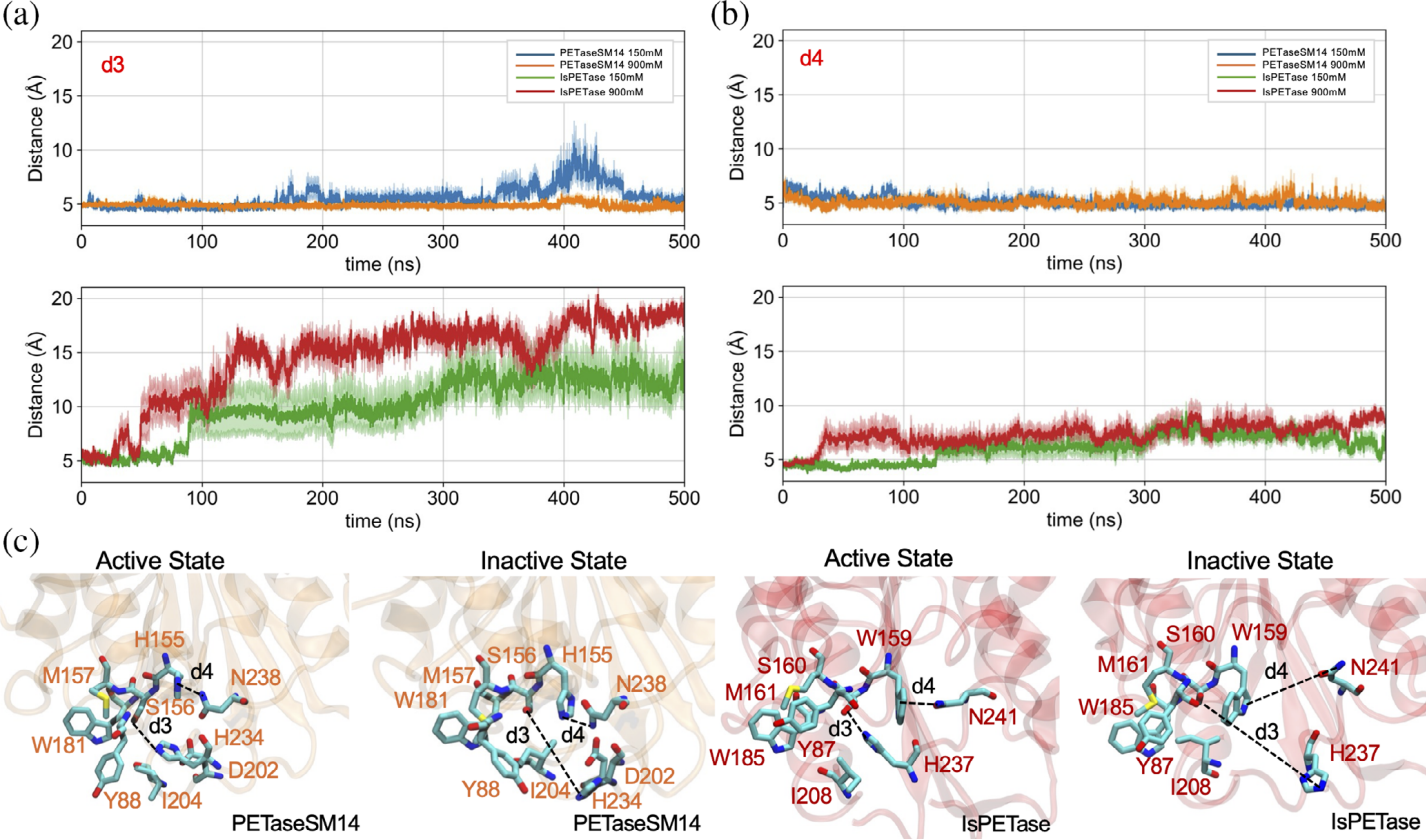
Catalytic triad stability in PETaseSM14 and IsPETase. The distances between (a) the catalytic serine and histidine side chains (d3) and between (b) H155 or W159, in PETaseSM14 and IsPETase, respectively, and the adjacent asparagine (d4) were calculated over the course of 500 ns‐long MD simulations. The value and the associated error are reported as the average and standard deviation over the three independent replicas performed for each system. (c) Representative snapshots of the active and inactive states of the PETaseSM14 and IsPETase active sites are shown.

To understand whether the different sizes of the enzymes' binding pockets at the two ion concentrations affect the integrity of the catalytic triad, we monitored the distance between the catalytic S160/S156 and H237/H234 side chains during the simulated trajectories (Figure 4a, d3). From this analysis, it can be observed that the d3 distance in PETaseSM14 is stable at both conditions. At 150 mM, values between 3.0 and 5.0 Å are reached (blue trace), with only a transient oscillation between 380 and 450 ns. Even greater stability is observed at 900 mM, at which d3 is confined between 2.5 and 3.0 Å for the first 400 ns, slightly increasing up to 5 Å only during the last part of the simulation (orange trace).

A different trend is observed for IsPETase. At 150 mM, the d3 distance is maintained stable below 5.0 Å for ~100 ns, then shifting to values up to 15.0 Å in the rest of the MD simulation (green trace). The disruption of the S160‐H237 interaction is observed at an earlier point in the trajectory, approximately 40 ns, at 900 mM. At that time, d3 starts to increase, reaching values of around 20 Å by the end of the simulation (red trace). Based on this evidence, when the catalytic S160/S156 interacts with H237/H234 (d3 ~ 5 Å), the enzyme is defined as *active*, because this configuration can initiate the hydrolysis of the PET chain (Figure 4c) (Austin et al., 2018; Berselli et al., 2024; Fecker et al., 2018). On the other hand, when d3 is disrupted, the enzyme becomes *inactive*, since the catalytic histidine is too far from the serine and the enzyme is not able to depolymerize the substrate even if it is correctly bound to the active site.

This structural rearrangement is associated with the remodeling and opening of the IsPETase binding sites. Indeed, we found that the disruption of d3 correlates with the stability of the interaction between the residue adjacent to the catalytic serine, W159 in IsPETase and H155 in PETaseSM14, with the conserved N241/N238 side chain (Figure 4b, d4). In particular, it can be observed that d4 remains stable in PETaseSM14 during the MD simulations, with average values of ~5.0 Å at both 150 mM (blue trace) and 900 mM (orange trace) of NaCl concentration, respectively. On the other hand, the same distance (d4) between W159 and N241 slowly increases over time for IsPETase at 150 mM of ion concentration (green trace), remaining stably below 5 Å for ~120 ns, then increasing up to ~6 Å for the successive 180 ns and ultimately reaching values of ~8 Å in the last part of the MD simulation. Moreover, the d4 interaction is lost more rapidly in the MD simulation performed at 900 mM of ion concentration (red trace), increasing from ~5 to ~7.5 Å within the first 40 ns, and eventually reaching ~9 Å by the end of the trajectory.

Notably, the displacement of W159 away from N241 results in the insertion of its indole side chain between S160 and H237 (Figure 4c). This rearrangement, which coincides with the transition from the active to the inactive state in IsPETase, prevents reformation of the catalytic contact, rendering the process largely irreversible within the simulated timescale. In contrast, in PETaseSM14, H155 remains stably bound to N238 throughout the simulations, regardless of NaCl concentration (Figure 4b, d4). This configuration allows S156 and H234 to align properly within the binding cleft (Figure 4c), maintaining the enzyme in an active conformation for approximately 85% of the simulation time at 150 mM NaCl and throughout the entire trajectory at 900 mM. Furthermore, unlike IsPETase, PETaseSM14 exhibits a reversible transition between active and inactive states, as shown by the temporary fluctuations of d3 (Figure 4a), which return to a stable value of ~5 Å for the remainder of the simulation.

### 2.5 PETaseSM14 binding site recruits more water at a high ion concentration

**FIGURE 5:**
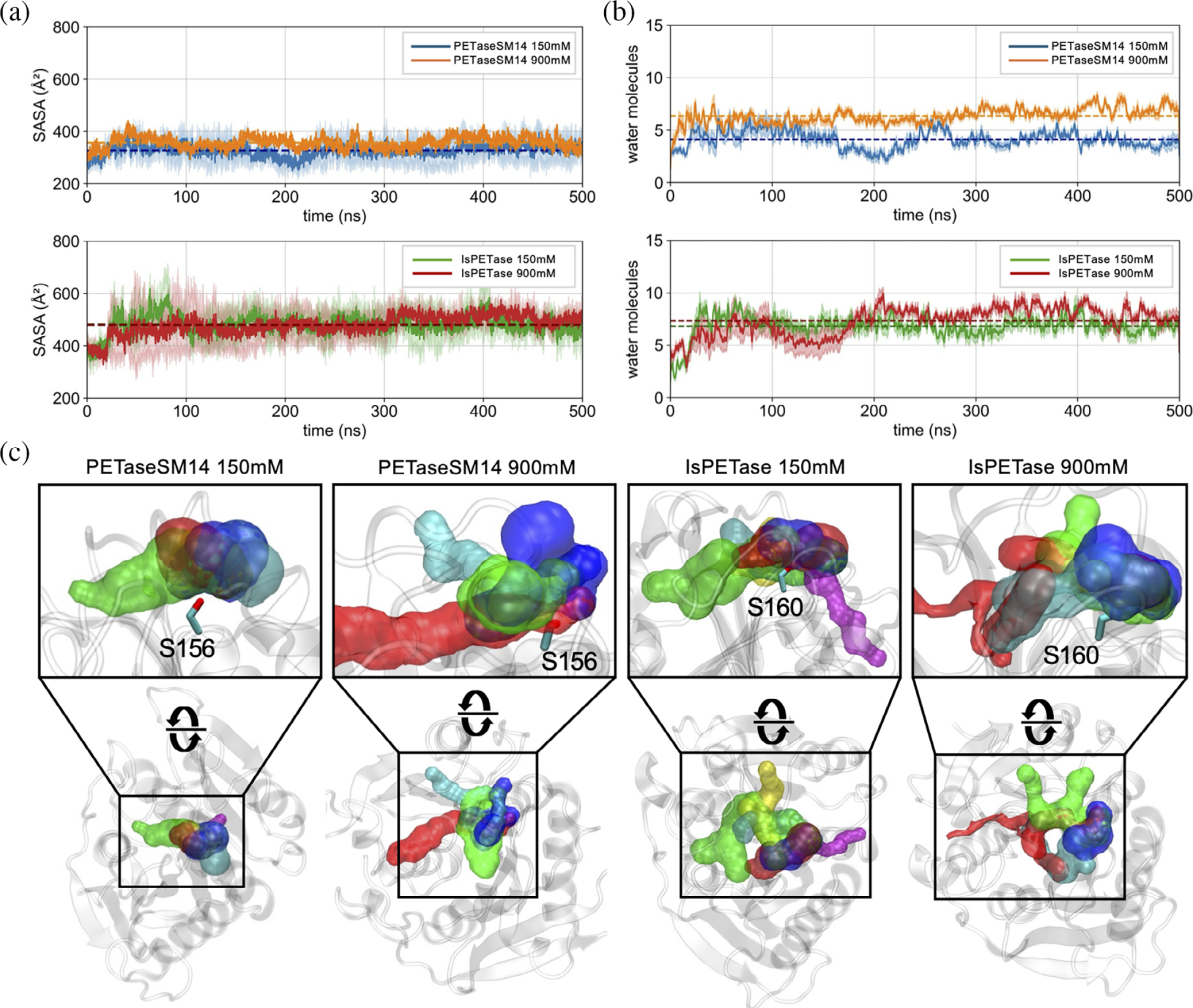
Hydration of the PETaseSM14 and IsPETase binding sites. (a) Solvent accessible surface area (SASA) and (b) number of water molecules inside the binding sites of PETaseSM14 (upper panels) and IsPETase (lower panels) during 500‐ns long MD simulations at 150 and 900 mM NaCl concentrations. The profiles and the associated errors are reported as the mean and standard deviation over the three replicas performed for each system. (c) Protein tunnels sampled near the active site with Caver (Chovancova et al., 2012). The most representative pathways obtained from clustering are shown in different colors. Calculations were initiated from the Oγ atom of the catalytic serine, whose side chain is depicted as sticks.

The changes in size and conformation of the binding pocket observed for the two enzymes are expected to influence their accessibility to both the solvent and the substrate. As an initial assessment of the binding site hydration, we calculated the solvent‐accessible surface area (SASA) of the enzymes' binding crevices (Figure 5a). This analysis showed that the solvent‐exposed surface area of PETaseSM14 is significantly lower than that found for IsPETase (Table S1). Interestingly, however, a modest yet significant ~10% increase in solvent‐exposed surface is observed for PETaseSM14 when the NaCl concentration increases from 150 to 900 mM. On the other hand, the SASA is almost identical in IsPETase at the two different conditions. This structural characteristic correlates with the number of water molecules that populate the cavity during the MD simulations (Figure 5b). This feature was calculated by adopting a 6 Å‐cutoff from the catalytic serine, which is buried at the bottom of the catalytic cleft and serves as the key site for the enzymatic activity. The average number of water molecules recruited by the binding site increases by ~35% in PETaseSM14 with raising ion concentration (Table S1). On the other hand, the binding site of IsPETase remains well hydrated under both conditions, with the number of water molecules changing by <10% as the ion concentration increases from 150 to 900 mM (Table S1). The variation in water‐accessible volume within the active sites of the two enzymes at different NaCl concentrations is highlighted by the tunnels sampled with Caver (Chovancova et al., 2012). In these analyses, tunnels originating near the active site were identified from conformations obtained during MD simulations. Figure 5c highlights the most representative pathways, selected after clustering and distinguished by different colors. Notably, in PETaseSM14 at 150 mM NaCl, the tunnel openings remain largely confined to the protein surface, with only minimal penetration into the enzyme interior. At 900 mM, however, although the superficial opening of the active site remains comparable to that at lower salt concentration, the accessible tunnels extend deeper into the active site, protruding further into the protein core. By contrast, in IsPETase at both 150 and 900 mM NaCl, the tunnels consistently span a broader surface volume and penetrate more extensively into the active site.

### 2.6 The increased ionic strength weakens the network of interactions within the binding site

**FIGURE 6:**
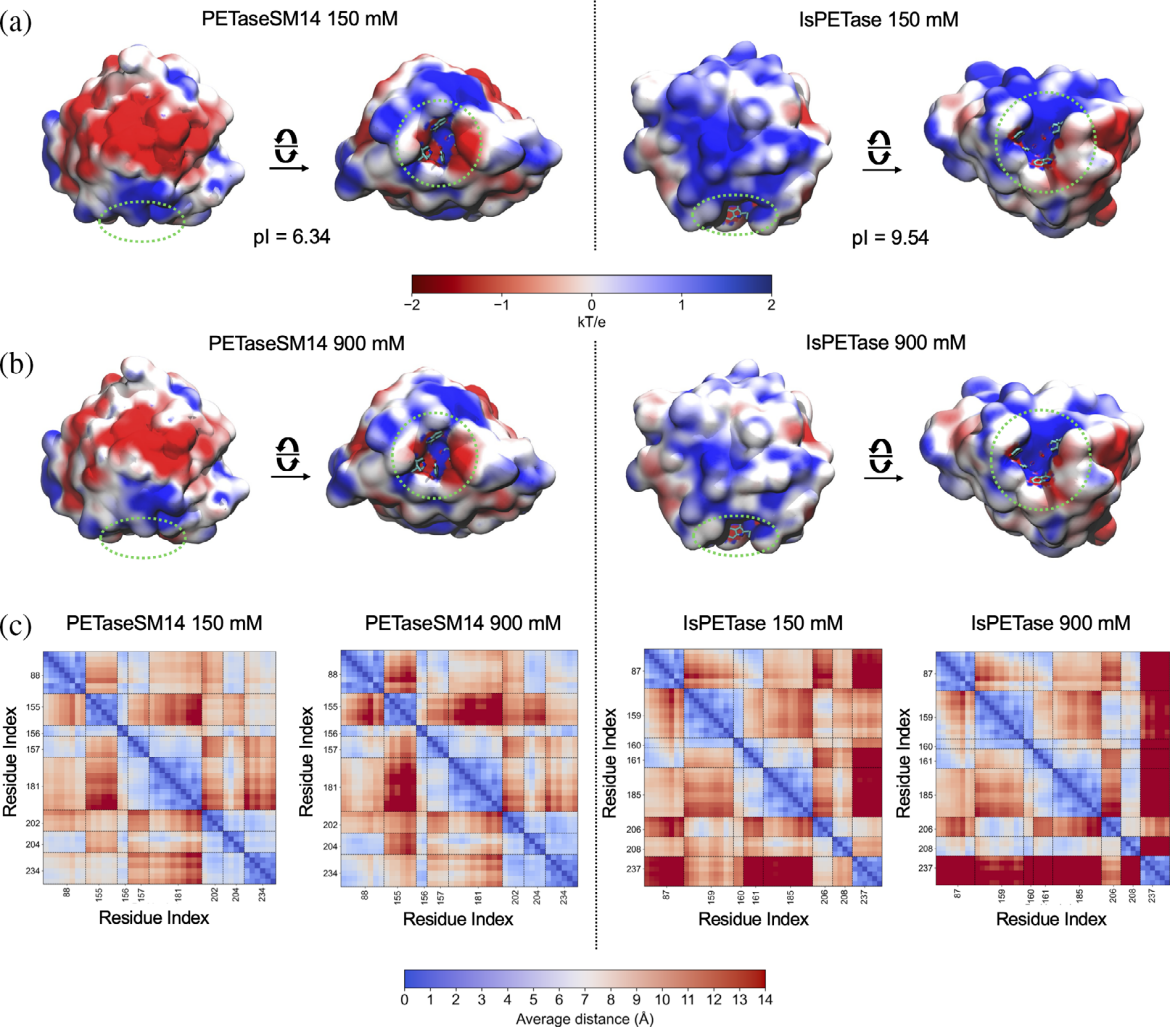
Effect of ion concentrations on the PETaseSM14 and IsPETase. Electrostatic potential surface of PETaseSM14 and IsPETase at (a) 150 mM and (b) 900 mM of NaCl concentration. The binding site locations and key residues are indicated with the green dotted circles. (c) Distance maps of residues forming the PETaseSM14 and IsPETase binding sites at the two ion concentrations. The maps report the average values of each cross‐distance calculated over the three 500‐ns MD simulation replicas. The color scale ranges from 0 Å (blue spots) to ≥14 Å (red spots).

The electrostatic potential surfaces of the two enzymes calculated at the two ion concentrations are reported in Figure 6a, b. At both conditions, PETaseSM14 exhibits a predominant acidic character (red regions) consistent with its lower isoelectric point (pI = 6.34) compared to IsPETase and establishing preferential contacts with cations (Figure S4a). On the other hand, IsPETase displays a basic character, with a pI = 9.54, and mainly interacts with anions (Figure S4b). However, the increase in the number of ions in solution to 900 mM (Figure 6b) reduces the extension of the charged surfaces of both PETaseSM14 and IsPETase in comparison to that observed at 150 mM (Figure 6a). This variation arises from the increasing number of interactions between the ions and the charged residues of the two enzymes, along with the decrease in the permittivity of the solvent under high‐salt conditions, which drops from ~73.6 at 150 mM to ~65.8 at 900 mM NaCl concentration (Buchner et al., 1999).

Interestingly, cation interactions occur predominantly on surface‐exposed protein regions that are distant from the active site of both enzymes (Figure S4c). Conversely, both PETaseSM14 and IsPETase form contacts with Cl^−^ near the active site, particularly at high‐salt concentrations (Figure S4d). This increase in protein‐anion interactions in the region surrounding the binding sites is expected to loosen the local stabilizing interaction network, particularly in IsPETase. To explore this hypothesis, we calculated the distances map between each residue of the binding site of the two enzymes (Figure 5c) as the average over values calculated from the simulated trajectories. By comparing the interactions pattern between residues in PETaseSM14 at the two ion concentrations, minimal variations are observed, with the distances between the pairs W155 and Y88, W155 and W181, W155 and I204, and M157 and H234 subjected to minimal increase at 900 mM. Importantly, the catalytic triad, composed of S156, H234, and D202, maintains inter‐residue distances consistently below 5 Å at both ionic strengths. This observation aligns with the *active*/*inactive* state transition described in Figure 4a, further supporting the structural integrity and conformational stability of the active site across varying ionic environments.

On the other hand, significant changes are observed in IsPETase. Even at 150 mM, the distances between residues belonging to the active site appear markedly larger than those observed in PETaseSM14, as pinpointed by the abundance of orange and red‐colored spots in Figure 5c, indicative of distances exceeding 9 Å. This pattern is consistent with the inherently broader and flexible binding site of IsPETase, which facilitates the efficient accommodation of the substrate under physiological conditions. Notably, at 150 mM, H237 is located more than 14 Å away from Y87, W185, and M161 (dark red regions), while some contacts are still observed with S160, W159 and D206 and I208. However, these interactions are entirely lost at 900 mM, owing to the rapid displacement of H237 that leads to the *inactivation* of IsPETase. This shift is accompanied by the movement of W159 towards D206 and I208 at 900 mM, which reflects the accelerated transition from the *active* to the *inactive* conformation of IsPETase under the high‐salt conditions previously described (Figure 4c).

### 2.7 PET‐bound *trans:gauche* ratio increases at the increase of ion concentration

Finally, we examined the interactions between the enzymes' active site and the PET chains localized at the surface of the slab. In order to characterize whether the model of amorphous PET employed in this work represented a realistic proxy of a physical sample, we calculated the distributions of the *trans* (Figure S5a) and *gauche* (Figure S5b) conformers in the bulk of the PET slab. Indeed, several experimental data showed that the percentage of the O–C–C–O dihedral angle corresponding to the *gauche* conformation (angle absolute value ~70°) ranges between 75% and 88% (Cunningham et al., 1974; Guévremont et al., 1995; Rodríguez‐Cabello et al., 1996; Wei et al., 2019), whereas the *trans* conformation (angle absolute value ~180°) corresponds to ~14% of the total distribution (Schmidt‐Rohr et al., 1998; Wei et al., 2019). Notably, the *trans:gauche* ratio calculated in the PET bulks of our MD simulations is around 14:82 in each system (Figure S6; distributions shown in Figure S5c,d), revealing a remarkable agreement with the data available in the literature (Cunningham et al., 1974; Guévremont et al., 1995; Rodríguez‐Cabello et al., 1996; Wei et al., 2019). However, when we considered only the fraction of PET bound to the active site of the enzymes, these distributions changed significantly. The monomers bound to PETaseSM14 at 150 mM are almost exclusively in the *gauche* conformation (Figure S6a), with only 0.26% of the *trans* state observed. On the other hand, in the PETaseSM14 at 900 mM (Figure S6b) and IsPETase at 150 mM (Figure S6c), the *trans:gauche* distribution closely resembles that observed in the bulk. Finally, in the IsPETase at 900 mM (Figure S6d), the ratio considerably drifts from that observed in the PET bulk, with ~32% of *trans* and only ~56% of *gauche* conformation.

These variations are attributed to the different sizes and accessibility of the enzymes' binding site at varying ion concentrations. At 150 mM, the binding cleft of PETaseSM14 remains relatively rigid and narrow. This prevents PET chains from being accommodated in the *trans* conformation, which requires more spatial freedom due to its extended geometry. Instead, at higher ion concentrations, the binding site of PETaseSM14 becomes capable of recruiting PET chains in a conformational distribution that mirrors that of the bulk phase.

A similar scenario is observed for IsPETase at 150 mM, whose naturally wider and more flexible binding cleft enables the recruitment of both *trans* and *gauche* PET conformers with bulk‐like statistics.

### 2.8 The hydrophobic and aromatic residues stabilize the PET binding onto the catalytic site

**FIGURE 7:**
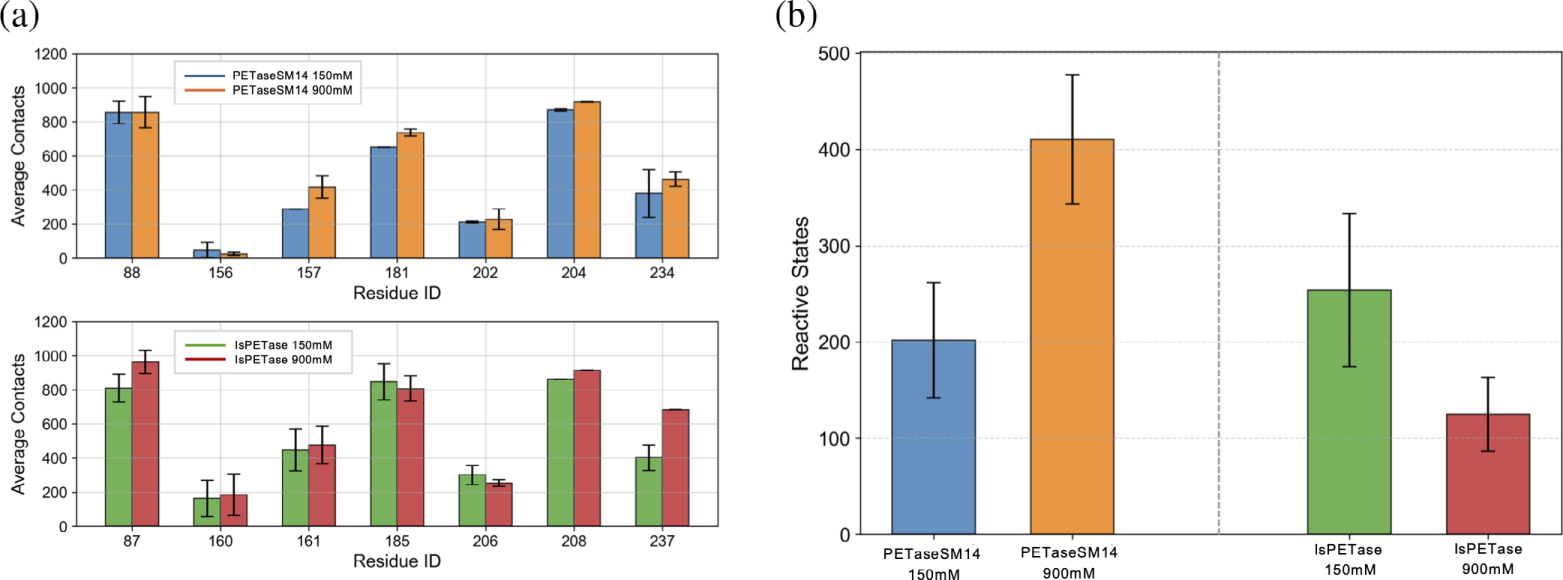
Interactions between PET and the PETaseSM14 and IsPETase binding sites. (a) Contacts between the PET chains and the residues forming the binding sites of PETaseSM14 (upper panel) and IsPETase (lower panel). The bars and the associated errors report the average and standard deviation of the total contacts established in each of the three 500‐ns‐long MD simulation replicas. (b) Number of *reactive* configurations sampled during the 500‐ns‐long MD simulations, averaged over the three replicas performed for each system.

A precise assessment of the contacts established by each residue of the enzyme's catalytic site with PET (Figure 7a) reveals that the most frequent interactions are provided by the aliphatic I208/I204 and the aromatic W185/W181 and Y87/Y88 side chains, collectively contributing to ~75% of the total enzyme‐PET contacts. In particular, these aromatic residues are known to play crucial roles in the substrate recognition and correct positioning within the active site by establishing stacking interactions with the benzene groups of the PET chains (Austin et al., 2018; Berselli et al., 2024). The time traces of the contacts formed by the side chains of W185/W181 and Y87/Y88 with the aromatic moieties of the PET chains (Figure S7) show distinct trends. For tryptophan, W185 in IsPETase forms approximately 20% more interactions with PET than W181 in PETaseSM14. Furthermore, the average values obtained at 900 mM are approximately 10% higher than those obtained at 150 mM for both enzymes. (Figure S7a). The average contact numbers for tyrosine in PETaseSM14 at 150 mM and 900 mM are slightly higher than in IsPETase at the same salt concentrations. Both systems establish ~15–20% more contacts at 900 mM than at 150 mM (Figure S7b). These results indicate that aromatic residues remain essential for coordinating substrate chains in both systems, with tryptophan contributing more contacts in IsPETase and tyrosine contributing more in PETaseSM14. Regarding the other residues of the binding site, a notable substrate exposure is observed for M161/M157 and H234/H237. In particular, the latter shows a significant 43% increase in the average contact number for IsPETase at 900 mM compared to 150 mM. This is consistent with the displacement of this residue towards the enzyme's surface during MD simulations, resulting in increased exposure to the substrate bulk (Figure 3a).

Finally, contacts between PET and the catalytic serine, which is buried at the bottom of the binding site, are the least frequent. Nevertheless, notable differences emerge between the two enzymes. Indeed, due to the broad and shallow architecture of the IsPETase binding site, PET chains establish approximately four times more interactions with S160 than with S156 in PETaseSM14 at both ion concentrations, indicating an improved capacity to accommodate the substrate near the catalytic center.

However, the trend changes significantly when considering the subset of configurations where the catalytic serine is simultaneously in contact with (i) one ester C‐atom of a PET chain and (ii) the enzyme binding site and the catalytic histidine (i.e., in the active state; Figure 7b). Under these conditions, with the enzyme in an active state (Figure 4) and effectively bound to the substrate, the system is primed for catalysis, and we refer to these configurations as *reactive* states. Based on statistics from our MD simulations, the average number of *reactive* states for PETaseSM14 is ~20% lower than those obtained for IsPETase at 150 mM (blue and green bar, respectively). However, at the higher NaCl concentration, the number of reactive states observed for IsPETase decreases by approximately 50% (Figure 7b, red bar), while that for PETaseSM14 doubles (Figure 7b, orange bar) relative to their respective values at 150 mM.

## 3 DISCUSSION

IsPETase, secreted by *Ideonella Sakaiensis* 201‐f6, and PETaseSM14, expressed by the marine sponge *Streptomyces* sp. SM14, are characterized by the same catalytic scaffold; however, significant differences have evolved to adapt each enzyme to its native environment. Activity assays on PET powder degradation performed in this study and supporting evidence from the literature (Carletti et al., 2025; Carr et al., 2023; Weigert et al., 2022) indicate that NaCl concentration exerts a positive effect on the activity of PETaseSM14, consistent with its hypersaline marine origin, while having a negative effect on IsPETase. To date, this phenomenon has been observed using analytical techniques such as HPLC, SEM, and AFM, which do not elucidate the structural basis of these effects.

To address this gap, we employed molecular modeling and MD simulations to deliver a detailed structural comparison and molecular‐level explanation of the experimentally observed differences between the two homologous plastic‐degrading enzymes. The results obtained from 500‐ns‐long MD simulations reveal that the IsPETase binding site is significantly wider and more flexible than that found for PETaseSM14. While this characteristic is beneficial for substrate binding at low ion concentration (150 mM), the excessive plasticity of the active site leads to the fast *inactivation* of IsPETase at a higher ion concentration (900 mM). The quick displacement of the catalytic residue H237 from S160 is triggered by the flipping of the W159 side chain, impairing the enzyme's ability to initiate hydrolysis, even when the substrate is correctly positioned within the binding site. The role of W159 in IsPETase has been assessed via site‐directed mutagenesis in a number of previous studies (Austin et al., 2018; Joo et al., 2018), in which this residue was replaced with a histidine. This mutation narrows the binding cleft, thus making the enzyme more structurally similar to other homologous cutinases. However, its impact on enzymatic activity remains a matter of debate. The bulky tryptophan side chain contributes to a shallower binding cleft compared to that of other hydrolases (Karunatillaka et al., 2022). While this characteristic is suggested by Joo et al. (2018) to facilitate substrate uptake and turnover, the W159H/S238F double variant characterized by Austin et al. (Austin et al., 2018) outperformed the WT enzyme in terms of crystalline PET degradation, suggesting a potentially different functional outcome.

By contrast, PETaseSM14 remains more rigid and undergoes minor, yet beneficial, conformational rearrangements during MD simulations at high ionic strength. Indeed, the slight opening of the binding crevice observed at 900 mM improves the recruitment of water and the substrate, while always maintaining the catalytic triad in an *active* state. In particular, under high‐salt conditions, the binding site of PETaseSM14 accommodates an average of ~2 additional water molecules compared to the low‐salt conformation, with the water‐accessible volume extending deeper into the active site relative to that observed at 150 mM. Given the mechanistic role of water in the catalytic mechanism of hydrolases (Berselli et al., 2025; Burgin et al., 2024; García‐Meseguer et al., 2023; Jerves et al., 2021), we suggest that the enhanced hydration of the PETaseSM14 binding site observed at high salinity contributes to sustained catalytic turnover.

To model PET binding, and consistent with a previous study on a similar system (Sahihi et al., 2024), we adopted 9‐monomers‐long PET chains to reproduce the amorphous polymer sample. Indeed, this size represents the minimal length to form an entangled network between polymeric chains, and it is considered an optimal trade‐off between accuracy in the reproduction of the mechanical features of amorphous PET and the computational cost (Aharoni, 1978; Roberge et al., 2004). The ability of PETaseSM14 at 900 mM and IsPETase at 150 mM to accommodate PET chains in the same conformational distribution observed in the bulk suggests greater substrate compatibility and potentially enhanced catalytic efficiency compared to PETaseSM14 at 150 mM or IsPETase at 900 mM. This is particularly relevant given that conformational transitions between *trans* and *gauche* states are extremely slow at room temperature (Wei et al., 2019), and the presence of a pre‐organized binding site capable of accommodating the substrate without requiring extensive conformational rearrangements could significantly enhance enzymatic turnover by making the process less entropically demanding.

Nevertheless, while the modest remodeling of the PETaseSM14 binding site does not substantially improve substrate penetration towards the catalytic serine, PET chains penetrate more efficiently in the broader and shallower cleft of IsPETase. Moreover, in both enzymes the substrate remains primarily stabilized by the surface‐exposed tryptophan (W181/W185) and tyrosine (Y88/Y87) side chains. This observation aligns with a number of previous studies highlighting the role of hydrophobic and π‐stacking interactions in PET stabilization (Austin et al., 2018; Berselli et al., 2024; da Costa et al., 2021; Fecker et al., 2018; Guo et al., 2023; Joo et al., 2018), as mutations in these residues were shown to be deleterious to the enzymatic activity of IsPETase (Austin et al., 2018; Han et al., 2017). However, the increased recruitment of water, the accommodation of PET chains with a bulk‐like conformational distribution and the persistent stability of the catalytic scaffold in an *active* state observed for PETaseSM14 at 900 mM, significantly enhances the number of *reactive* configurations sampled during the MD simulations. Indeed, the states in which the enzyme is simultaneously in an *active* form and the PET chain is correctly positioned within the binding site of PETaseSM14 at 900 mM occur at approximately twice the frequency compared to 150 mM NaCl. In contrast, IsPETase exhibits approximately a 50% reduction in reactive states when transitioning from 150 to 900 mM NaCl. This trend mirrors the experimentally measured activity pattern, bridging molecular simulations insights with in vitro physiological responses and providing a molecular‐level explanation of the structural adaptations that enable enzymatic activity across diverse environments.

The findings of this study help pave the way for precise engineering strategies aimed at enhancing the effectiveness of these biotechnologies in marine ecosystems, where the accumulation of micro‐ and nanoplastics is an urgent environmental concern.

## 4 MATERIALS AND METHODS

### 4.1 Production of PETases

The enzymes employed in this work were produced in accordance with previous studies (Carletti et al., 2025; Di Rocco et al., 2023). IsPETase from *Ideonella sakaiensis* 201‐f6 was expressed and purified from the green microalga *Chlamydomonas reinhardtii*. We used a photosynthetic restoration strategy in which the enzyme is constitutively expressed in the chloroplast, as reported by Di Rocco et al. (2023). PETaseSM14 from the marine sponge *Streptomyces* sp. SM14 was produced in *Escherichia coli* BL21 (DE3) and purified as reported by Carletti et al. (2025).

### 4.2 Enzyme activity assay

All chemicals used were of the highest purity available. All aqueous solutions were prepared in deionized water. For the TPA calibration curve reported in Figure S2 a pure TPA powder from Fluka chemicals (n.86420, purity >99%) was used. The enzymatic activity was evaluated using PET powder (Goodfellow, product code ES30‐PD‐000132, particle size 300 μm, crystallinity >50%) at the final concentration of 5 mg mL^−1^, into 100 μL buffer containing 1 μM protein solution. Thermal and pH conditions assessed in previous works have been used (Carletti et al., 2025; Di Rocco et al., 2023), in particular: 100 mM Tris–HCl buffer at pH 8.0 for IsPETase and 100 mM Tris–HCl pH 9.0 for PETaseSM14, incubated at 37°C. The NaCl concentrations tested were 0, 150, 300, 700 and 900 mM. After an incubation time of 72 h, the reaction tubes were vigorously mixed and centrifuged, then the supernatant was filtered and further analyzed by RP‐HPLC. For every set of reactions, two control samples were prepared following the same procedure: one without adding the protein, while the other without adding the substrate. The RP‐HPLC analysis consists of a linear gradient procedure using an Agilent Poroshell 120 EC‐C18 column, equilibrated with a mobile phase of 80:20 solution A (0.1% formic acid): solution B (100% acetonitrile). 20 μL of each sample were loaded into the column and eluted over a 20‐min run at a flow rate of 1 mL/min, at room temperature with the following elution steps: 80:20 (solution A: solution B), followed by a 15‐min linear gradient 20:50 (solution A: solution B), 2 min isocratic 50:50 (solution A: solution B), followed by 3 min linear gradient from 50:20 (solution A: solution B) and 2 min isocratic 80:20 (solution A: solution B). The absorbance was measured at 240 nm and 254 nm, to detect the carbonyl groups and aromatic rings of the reaction products. To determine peak areas, the baseline was drawn manually and calculated using instrument software. According to the chromatograms obtained (Figure S8), the reaction product with the highest retention time was BHET (2.9 min), followed by MHET (2.5 min, assumed) and TPA (1.7 and 2.1 min), which was the main product obtained.

### 4.3 Preparation and equilibration of PET9 slab

The initial configuration and topology of the PET9 melt system was produced with the *polymer builder* tool of CHARMM‐GUI (Figure S9a) (Choi et al., 2021; Jo et al., 2008). We generated a cubic system with size ~60 × 60 × 60 Å^3^ and including 100 9‐monomers‐long PET chains, resulting a total of 20,000 atoms, and a density of ~1.23 g · cm^−3^, in line with the experimental value (Thompson & Woods, 1955). This system was parametrized using the CHARMM general force field (CGenFF) (Vanommeslaeghe et al., 2010), which demonstrated successful performances in previous works on ion track formation in PET samples (Shen et al., 2023), ion conduction through PET nanopores (Cruz‐Chu et al., 2009), and PET binding onto plastic degrading enzymes (Berselli et al., 2024; Polêto & Lemkul, 2025; Sahihi et al., 2024). Following the protocol prescribed by CHARMM‐GUI, the coarse‐grained (CG) model of the melted PET systems was equilibrated at 300 K, followed by the conversion to the all‐atom structure, that is provided as an output to the user. This represents the starting structure for classical MD simulations, which was further minimized for 10,000 steps followed by 250 ps of equilibration in the NVT ensemble at a temperature of 300 K and the heavy atoms of the system restrained. Then, the system underwent a simulated annealing procedure to achieve an amorphous‐like conformational distribution in the absence of positional restraints. This step was conducted by progressively heating the system from 300 to 750 K with a 50 K · ps^−1^ rate. Then, 4 ps of equilibration were carried out at 750 K, followed by cooling from 750 to 300 K with the same rate as that used for heating. We stress that this procedure did not require extensive simulations to achieve an accurate representation of amorphous PET sample thanks to the reliable initial configuration provided by CHARMM‐GUI. After the simulated annealing step, further 250 ps of equilibration were carried out in the NVT ensemble at *T* = 300 K, followed by 1 ns of standard MD simulation in NPT at *T* = 300 K and *p* = 1 bar. Constant temperature and pressure were maintained by a Langevin thermostat and Nosé‐Hoover Langevin barostat, respectively (Feller et al., 1995; Martyna et al., 1994). The oscillation piston period was set to 50.0 fs and the damping time scale to 25.0 fs. The damping coefficient of the Langevin thermostat was set to 1 ps^−1^. Long‐range electrostatic interactions were computed using the Particle Mesh Ewald (PME) algorithm (Darden et al., 1993), with spline interpolation order 6. Electrostatic and van der Waals (VdW) interactions were calculated with a cutoff of 12 Å as prescribed by the CHARMM force field. A switching function was applied, starting to take effect at 10 Å to obtain a smooth decay as indicated in Ref. (Steinbach & Brooks, 1994). Chemical bonds involving hydrogen atoms and heavy atoms were constrained with SHAKE (Ryckaert et al., 1977), enabling the adoption of a time step (δ t) of 2 fs. Each MD simulation was performed with NAMD (Phillips et al., 2005; Phillips et al., 2020).

### 4.4 Assembly of enzyme‐PET systems

To reproduce the system with the enzyme adsorbed onto the PET surface, we selected as a starting configurations the crystallographic structures of the IsPETase (PDB ID: 6EQE, resolution: 0.92 Å) (Austin et al., 2018) and PETaseSM14 (PDB ID: 9HYD, resolution: 1.43 Å) (Carletti et al., 2025). The CHARMM coordinate file for these structures were generated with the PDB reader of CHARMM‐GUI (Jo et al., 2008), by specifying the protonation state of the histidine residues based on the pKa predicted with PropKa (Olsson et al., 2011). Then, using VMD (Humphrey et al., 1996), we manually positioned the enzyme structures and the equilibrated model of the melted PET within the same simulation box, with the catalytic serine (S160 in IsPETase, S156 in PETaseSM14) pointing towards the PET surface. At the initial stage, the enzyme and PET surface were kept separated by a minimal distance of 7 Å to prevent steric clashes and significant interactions (Figure S9b). The initial configurations of the enzyme‐PET systems were solvated and added with the NaCl ion bath (150 or 900 mM) using the *solvate* and *autoionize* packages of VMD, respectively, while the topologies were built with *psfgen*. The ester bond between the nine PET monomers belonging to the same chain were preserved by adding the patch P00080. Moreover, the disulfide bonds between C203 and C239 and between C273 and C289 of IsPETase were included. The resulting solvated systems were included in rectangular boxes with size 90 × 90 × 150 Å^3^, counting approximately 110,000 atoms each (Figure S9c).

### 4.5 Molecular dynamics simulations

The solvated enzyme‐PET systems at the two different ion concentrations were equilibrated following a multi‐step procedure. After 5 ps of energy minimization, the system was progressively heated to the selected temperature of 310 K. Then, 10 ns of equilibration were conducted in the NVT ensemble adopting a δ t = 1 fs, followed by 20 ns in NVT with δ t = 2 fs and 20 ns in NPT with δ t = 2 fs. This cumulative 50‐ns‐long equilibration was performed with progressive release of positional constraints to allow slow relaxation of the system and the diffusion of PET chains, allowing the adsorption of the enzyme onto the PET surface (Figure S9b). The timestep, timescale, ensemble and restraints adopted in each of the five 10‐ns‐long steps of equilibration are summarized in Table S2.

After the equilibration, 500 ns of standard MD simulations were performed maintaining only the minimal restraints as those used in the last step of equilibration to avoid roto‐translation of the protein during the MD simulation (Figure S10). To ensure adequate statistics and reproducibility, each system was separately equilibrated and simulated in three independent replicas. The topology, coordinate and output files associated with each replica of each system (IsPETase‐PET9 and PETaseSM14‐PET9 at 150 and 900 mM of NaCl concentration) are freely available on Zenodo (https://zenodo.org/records/17412459). Rectangular PBCs were adopted to replicate the system and remove box surface effects. Chemical bonds involving hydrogen atoms and heavy atoms of the protein were constrained with SHAKE (Ryckaert et al., 1977), while those of water molecules were kept fixed with SETTLE (Miyamoto & Kollman, 1992). Each MD simulation was performed with NAMD (Phillips et al., 2005; Phillips et al., 2020) and the CHARMM36/CHARMM36m (Huang et al., 2017; Huang & MacKerell, 2013) force field under Langevin dynamics adopting the same parameters as those used for melted PET equilibration.

### 4.6 Analysis of molecular dynamics simulations

#### 4.6.1 RMSD and RMSF

The backbone root‐mean‐square deviations (RMSD), the root‐mean‐square fluctuations (RMSF) of the enzyme, the inter‐residue distances, and the inter‐residue distance maps were calculated with MDAnalysis (Gowers et al., 2016; Michaud‐Agrawal et al., 2011) and plotted with the *matplotlib* library of Python (https://matplotlib.org) (Hunter, 2007). The results and associated error are reported as the average and standard deviation over the three replicas performed for each system.

#### 4.6.2 Inter‐residue distances

The distances calculated to assess the size of the active site cleft (d1, d2, Figure 3) or the active/inactive states (d3, d4, Figure 4) were calculated with MDAnalysis (Gowers et al., 2016; Michaud‐Agrawal et al., 2011), and reported as the mean and standard deviation over the three replicas performed for each system. The atoms considered in each calculation are indicated in Table 1.

#### 4.6.3 SASA

The solvent‐accessible surface area (SASA) of the binding sites of the protein (S156/S160, H234/H237, D204/D206, M157/M161, Y88/Y87 and W181/W185 in PETaseSM14/IsPETase) was calculated with VMD (Humphrey et al., 1996) using a probe radius of 1.4 Å.

#### 4.6.4 Water molecules inside the binding pocket

The number of water molecules inside the binding pocket was calculated with MDAnalysis (Gowers et al., 2016; Michaud‐Agrawal et al., 2011) by considering a radius of 6 Å from the catalytic serine Oγ atom. This cutoff was selected as this is the distance between the catalytic serine Oγ atom and the Oγ atom of the Y87/Y88 side chain, which is the outermost, solvent‐exposed residue of the binding site (Figure S11).

#### 4.6.5 Protein tunnels

The tunnels and the free volume near the active site of the system were sampled with Caver 3.0 (Chovancova et al., 2012). The Oγ atom of the catalytic serine was used as the starting point for the calculations. Configurations were sampled every 50 ns along each MD trajectory using the default parameters and the resulting tunnels were clustered using the average‐link hierarchical algorithm with a threshold of 4.0.

#### 4.6.6 APBS

The electrostatic potential maps were computed with the adaptive Poisson‐Boltzmann solver (APBS) code (Jurrus et al., 2018). The crystal structures of the two enzymes were used as input for the APBS calculations. Prior to the calculations, the NaCl concentrations and the solvent dielectric constants were set to match the conditions of the MD simulations. The dielectric constants were chosen based on the values reported in Ref. (Buchner et al., 1999) for the temperature *T* = 308 K, with 73.57 and 65.80 being the closest available values for the target concentrations of 150 and 900 mM, respectively.

#### 4.6.7 Isoelectric point

The isoelectric point (pI) of each protein was calculated with the ExPASy Analysis tool (Gasteiger et al., 2005).

#### 4.6.8 Contacts number

The contacts between ions and the enzyme's surface were calculated with MDAnalysis (Gowers et al., 2016; Michaud‐Agrawal et al., 2011) adopting a cutoff of 5 Å.

#### 4.6.9 PET chains conformational distribution

The conformational distribution of the PET chains was calculated by considering the dihedral angles formed by the ethylene glycol unit of each monomer, composed of the atoms O3–C9–C10–O1, according to the PDB nomenclature used in the coordinate and topology files. Following the classifications proposed in Refs (Alves et al., 2002; Wei et al., 2019). the conformation was defined as *gauche* when the absolute value of the dihedral angle was 70° ± 20°, while it was defined as *trans* when the absolute value of the dihedral angle was 180° ± 20°. Dihedral angles of the substrate bound to the enzyme's binding site were calculated from the PET fraction within 8 Å of the catalytic serine, while those for the substrate bulk were obtained from the 100 PET chains excluding the bound fraction.

#### 4.6.10 Number of enzyme‐PET reactive states

The number of *reactive* states was calculated with MDAnalysis (Gowers et al., 2016; Michaud‐Agrawal et al., 2011) by counting the number of states in which the distance between the O γ atom of the catalytic serine side chain was simultaneously <5 Å away from the N ε atom of the catalytic histidine and from the ester carbon atom of a PET chain (C1 or C8 according to the PDB nomenclature used in the coordinate and topology files). The results and associated errors are reported as the average and standard deviation over the three replicas.

## AUTHOR CONTRIBUTIONS

**Alessandro Berselli:** Conceptualization; investigation; writing – original draft; methodology; validation; visualization; writing – review and editing; formal analysis; project administration; data curation; resources. **Alan Carletti:** Conceptualization; investigation; writing – review and editing; visualization; methodology; data curation. **Maria Cristina Menziani:** Conceptualization; investigation; writing – review and editing; project administration; supervision; funding acquisition; resources. **Shapla Bhattacharya:** Writing – review and editing; validation. **Rossella Castagna:** Validation; writing – review and editing. **Emilio Parisini:** Writing – review and editing; validation; conceptualization. **Giulia di Rocco:** Conceptualization; supervision; writing – review and editing; visualization; validation; methodology; investigation; project administration. **Francesco Muniz‐Miranda:** Project administration; funding acquisition; investigation; conceptualization; writing – review and editing; visualization; methodology; supervision; resources.

## CONFLICT OF INTEREST STATEMENT

The authors declare no conflict of interest.

## Supporting information


**FIGURE S1.** Sequence alignment of PETaseSM14 and IsPETase.
**FIGURE S2.** TPA calibration curve.
**FIGURE S3.** RMSD and RMSF of PETaseSM14 and IsPETase.
**FIGURE S4.** Average contacts between the enzymes and ions.
**FIGURE S5.** *Trans:gauche* conformational distribution of PET chains.
**FIGURE S6.** Conformational distribution of PET chains.
**FIGURE S7.** Contact number between the aromatic side chains within the active and the PET benzene groups.
**FIGURE S8.** PETase reaction products released at different salt concentrations.
**FIGURE S9.** Enzymes adsorbed onto PET slabs simulation systems.
**FIGURE S10.** Positional restraints applied to the enzymes.
**FIGURE S11.** Definition of the cutoff for water molecules in the active site.
**TABLE S1.** Solvation of the enzymes' binding site from molecular dynamics simulations.
**TABLE S2.** Equilibration phase of the enzymes adsorbed onto the PET slab systems.

## Data Availability

All MD simulations were produced with NAMD3 (https://www.ks.uiuc.edu/Research/namd/). Trajectories were analyzed with VMD (https://www.ks.uiuc.edu/Research/vmd/) and the Python library MDAnalysis (https://www.mdanalysis.org). NAMD (https://www.ks.uiuc.edu/Research/namd/license.html) and VMD (https://www.ks.uiuc.edu/Research/vmd/current/LICENSE.html) are distributed free of charge for non‐exclusive, non‐commercial use. MDAnalysis is available under the “GNU general public license” (https://www.gnu.org/licenses/old-licenses/gpl-2.0.html). Data for PET slab equilibration, initial structure and topology of each PETase‐PET system at two ion concentrations and intermediate coordinate, input and output files (every 10 ns) for the three 500‐ns‐long replicas of each system and the HPLC data analysis are publicly available on Zenodo. https://zenodo.org/records/17412459. Each additional file can be provided by the authors upon reasonable request.
