# Supplementary material for: The effect of ionic strength on PETase enzymes: An experimental and computational study
Source: Protein Sci. 2025 Dec 27;35(1):e70386. doi: 10.1002/pro.70386 (PMC12743318; doi:10.1002/pro.70386)
Supplement: Supplementary file 1 — FIGURE S1. Sequence alignment of PETaseSM14 and IsPETase. FIGURE S2. TPA calibration curve. FIGURE S3. RMSD and RMSF of PETaseSM14 and IsPETase. FIGURE S4. Average contacts between the enzymes and ions. FIGURE S5. Trans:gauche conformational distribution of PET chains. FIGURE S6. Conformational distribution of PET chains. FIGURE S7. Contact number between the aromatic side chains within the active and the PET benzene groups. FIGURE S8. PETase reaction products released at different salt concentrations. FIGURE S9. Enzymes adsorbed onto PET slabs simulation systems. FIGURE S10. Positional restraints applied to the enzymes. FIGURE S11. Definition of the cutoff for water molecules in the active site. TABLE S1. Solvation of the enzymes' binding site from molecular dynamics simulations. TABLE S2. Equilibration phase of the enzymes adsorbed onto the PET slab systems. [file PRO-35-e70386-s001.pdf]

Supporting Information for:

The effect of ionic strength on PETase enzymes: an experimental and computational study

Alessandro Berselli[1‡], Alan Carletti[2], Maria Cristina Menziani[1], Shapla Bhattacharya[3,4], Rossella Castagna[3,5], Emilio Parisini[3,6], Giulia Di Rocco[2], Francesco Muniz-Miranda[1‡]

[1] *Department of Chemical and Geological Sciences (DSCG), University of Modena and Reggio Emilia (UNIMORE), Via Campi, 103, 41125 Modena, Italy*
[2] *Department of Life Sciences (DSV), University of Modena and Reggio Emilia (UNIMORE), Via Campi 103, 41125 Modena, Italy*
[3] *Department of Biotechnology, Latvian Institute of Organic Synthesis, Aizkraukles 21, LV-1006 Riga, Latvia*
[4] *Faculty of Natural Sciences and Technology, Riga Technical University, Paula Valdena 3, LV-1048 Riga, Latvia*
[5] *Department of Chemistry, Materials and Chemical Engineering "G. Natta", Politecnico di Milano, Piazza Leonardo da Vinci 32, 20133 Milano, Italy*
[6] *Department of Chemistry "G. Ciamician", University of Bologna, Via P. Gobetti 85, 40129 Bologna, Italy*

Authors:
Alan Carletti: alan.carletti@unimore.it ORCID: 0009-0009-5353-865X
Maria Cristina Menziani: mariacristina.menziani@unimore.it ORCID: 0000-0003-3428-5297
Giulia Di Rocco: giulia.dirocco@unimore.it ORCID: 0000-0002-3187-2210
Shapla Bhattacharya: shapla.bhattacharya@osi.lv ORCID: 0000-0002-1286-5138
Rossella Castagna: rossella.castagna@osi.lv ORCID: 0000-0002-9284-3165
Emilio Parisini: emilio.parisini@osi.lv ORCID: 0000-0002-5529-0039

[‡] Corresponding authors:
Alessandro Berselli: alessandro.berselli@unimore.it ORCID: 0000-0002-2241-3530
Francesco Muniz-Miranda: francesco.munizmiranda@unimore.it ORCID: 0000-0002-7614-2326

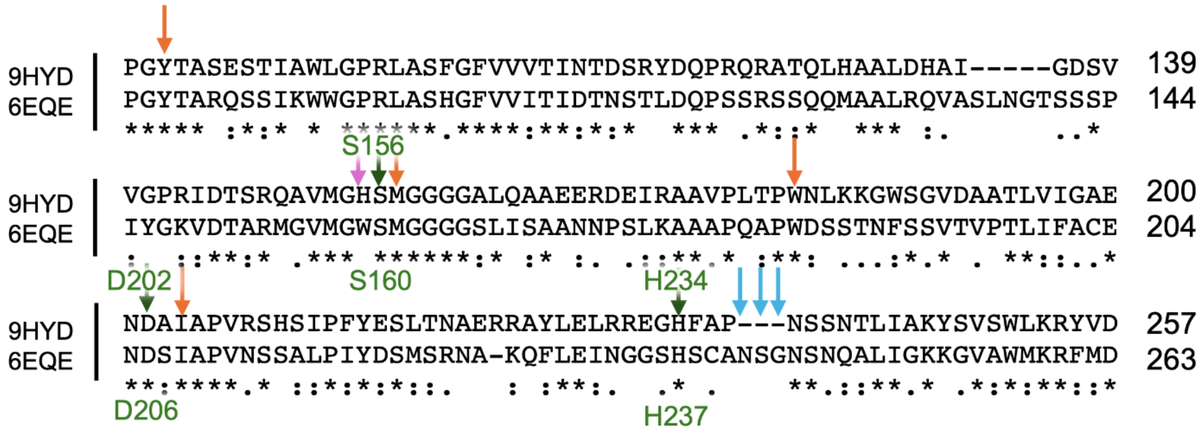

Figure S1. Sequence alignment of PETaseSM14 (PDB ID: 9HYD) and IsPETase (PDB ID: 6EQE). Catalytic triad residues are indicated by green arrows, while the remaining binding site residues are shown in orange. The H155/W159 residue in PETaseSM14 and IsPETase is marked with a pink arrow, and the three-residue loop extension unique to IsPETase is highlighted in cyan.

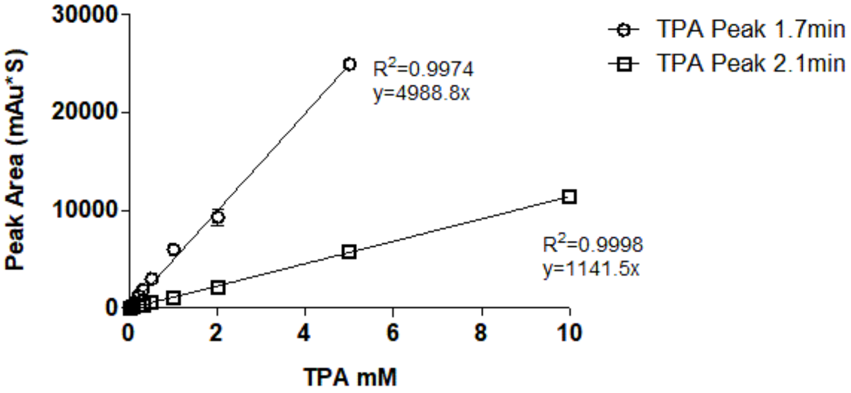

Figure S2. TPA calibration curve. The data were collected at 254 nm wavelength with the same analytical method reported in the materials and methods. Each point was generated by a triplicate measurement (n=3), the error bars when not visible fall within the size of the symbols. As reported in Figure S8, the TPA powder generates two different peaks, one at 1.7 min and one at 2.1 min; y = 4988.8x and y = 1141.5x, respectively. Regardless of the batch of pure TPA powder used, the results obtained were consistent with those reported here and depended on the solvent employed. When TPA was dissolved in pure DMSO, a single peak appeared at 2.1 min. Conversely, in the reaction buffers (pH 8 and 9), two peaks were detected, at 1.7 min and 2.1 min, corresponding to those observed in the reaction samples (**Figure S8**). Terephthalic acid (TPA) contains two carboxylic acid groups that remain fully protonated in DMSO, resulting in a single peak at 2.1 min. Moreover, solution A (0.1% formic acid) has a pH of 2.6, which further stabilizes the protonated form of TPA. Under basic conditions TPA tends to deprotonate, increasing its polarity and leading to the appearance of two distinct peaks, representing a mixture of protonated and deprotonated species. For quantification, reported in **Figure 2**, only the peak at 2.1 min was used to estimate the amount of TPA released. Although this approach slightly underestimates the total TPA amount released from the reactions, it provides a reliable basis for comparing the activity of the two enzymes.

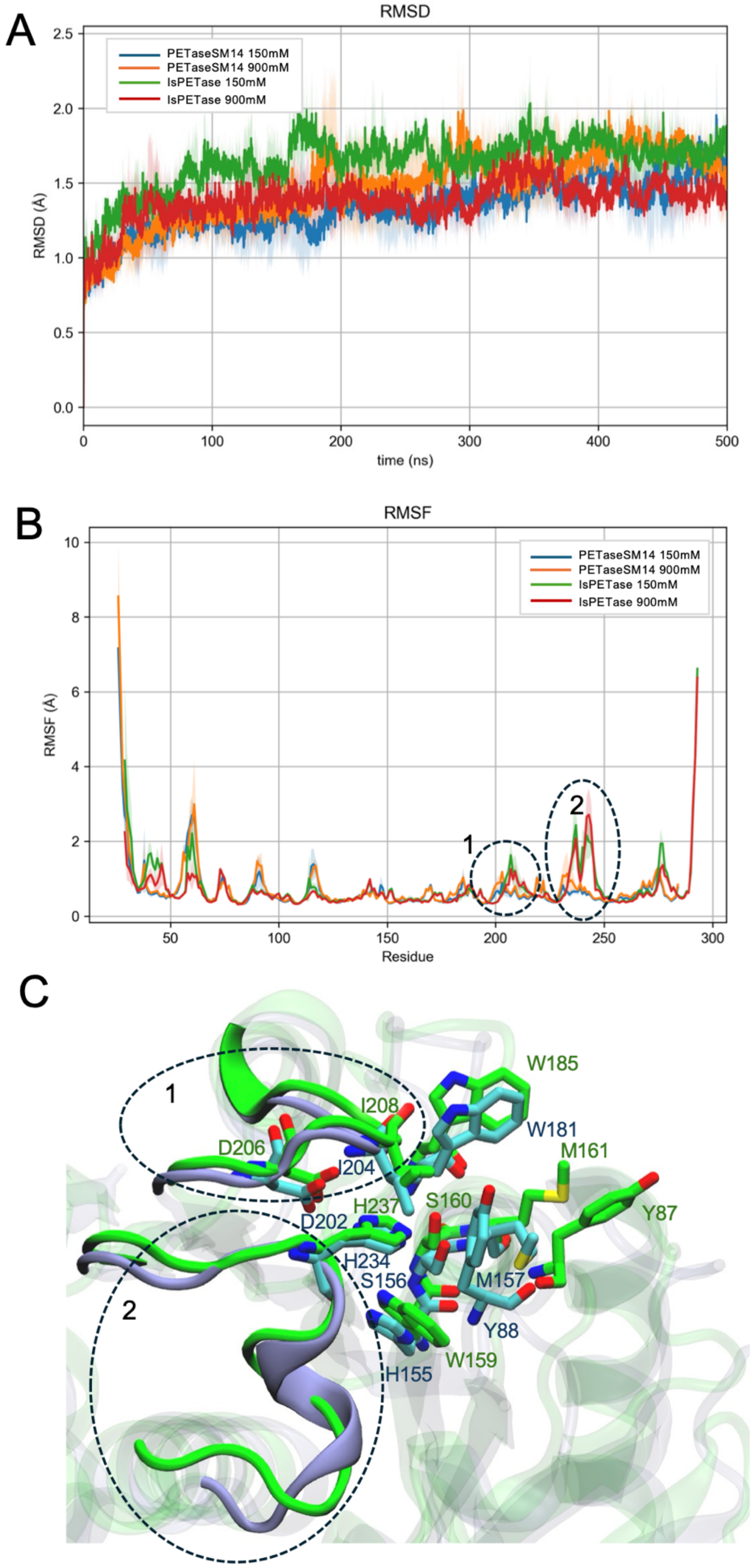

Figure S3. RMSD and RMSF of PETaseSM14 and IsPETase. (**A**) RMSD and (**B**) RMSF of PETaseSM14 and IsPETase at 150 mM and 900 mM of NaCl concentration. The profiles and the associated errors are reported as the mean and standard deviation over the three 500-ns MD simulation replicas. (**C**) Superposition of the PETaseSM14 (blue) and IsPETase (green) binding sites. The high flexibility domains surrounding the IsPETase binding sites are highlighted with the black dotted circles.

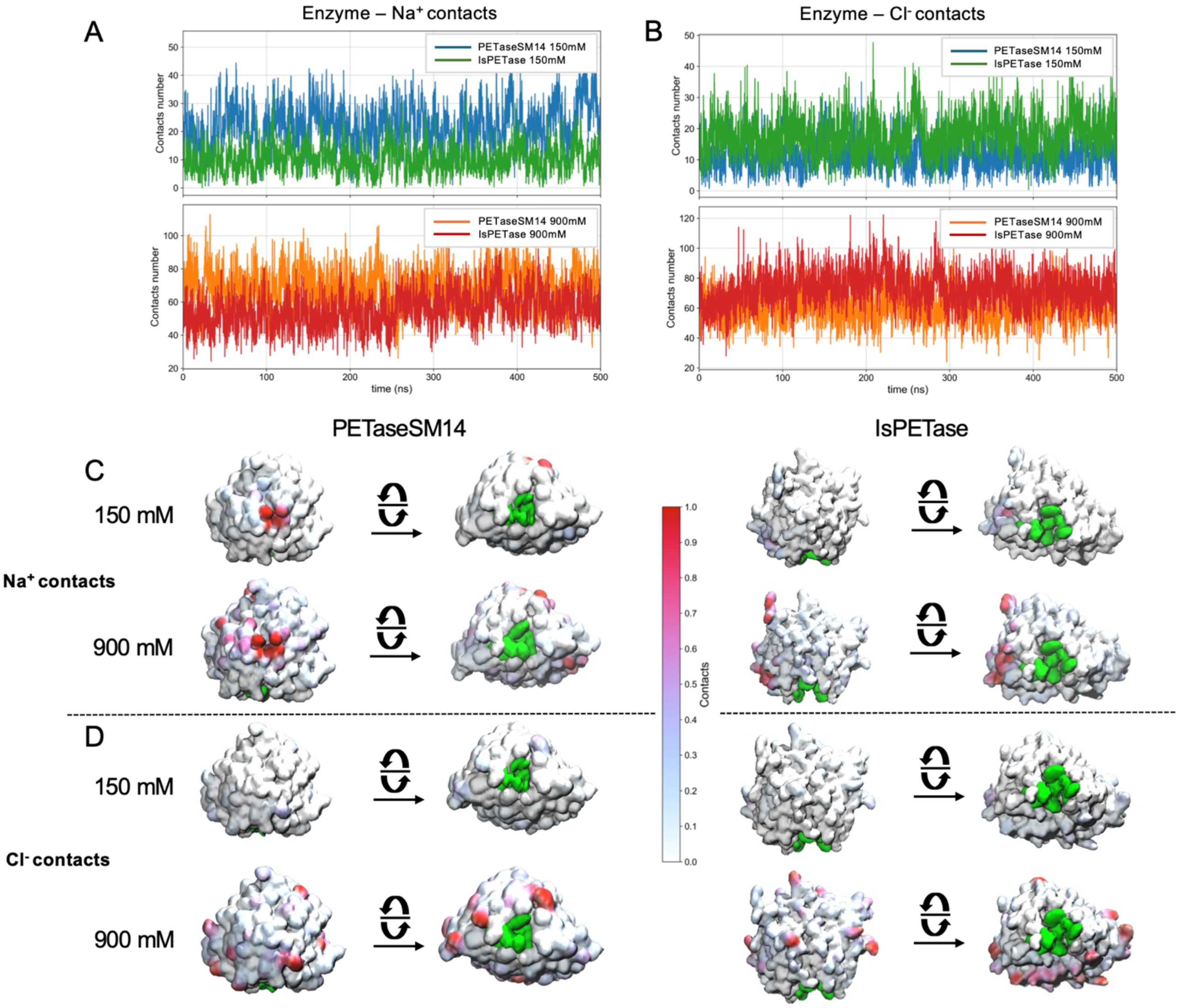

Figure S4. Average contacts between the enzymes and ions. Total contacts number between the PETaseSM14 and IsPETase and (**A**) Na^+ ions and (**B**) Cl^- ions at 150 mM (upper panels) and 900 mM (lower panels) of NaCl concentrations. The profiles report the average values during standard 500-ns-long MD simulations calculated over the three replicas performed for each system. Crystallographic structures of PETaseSM14 and IsPETase colored as a function of the contacts between the amino acids of each enzyme and (**C**) Na^+ and (**D**) Cl^- ions, averaged over the three replicas. The position of the binding site is highlighted in green.

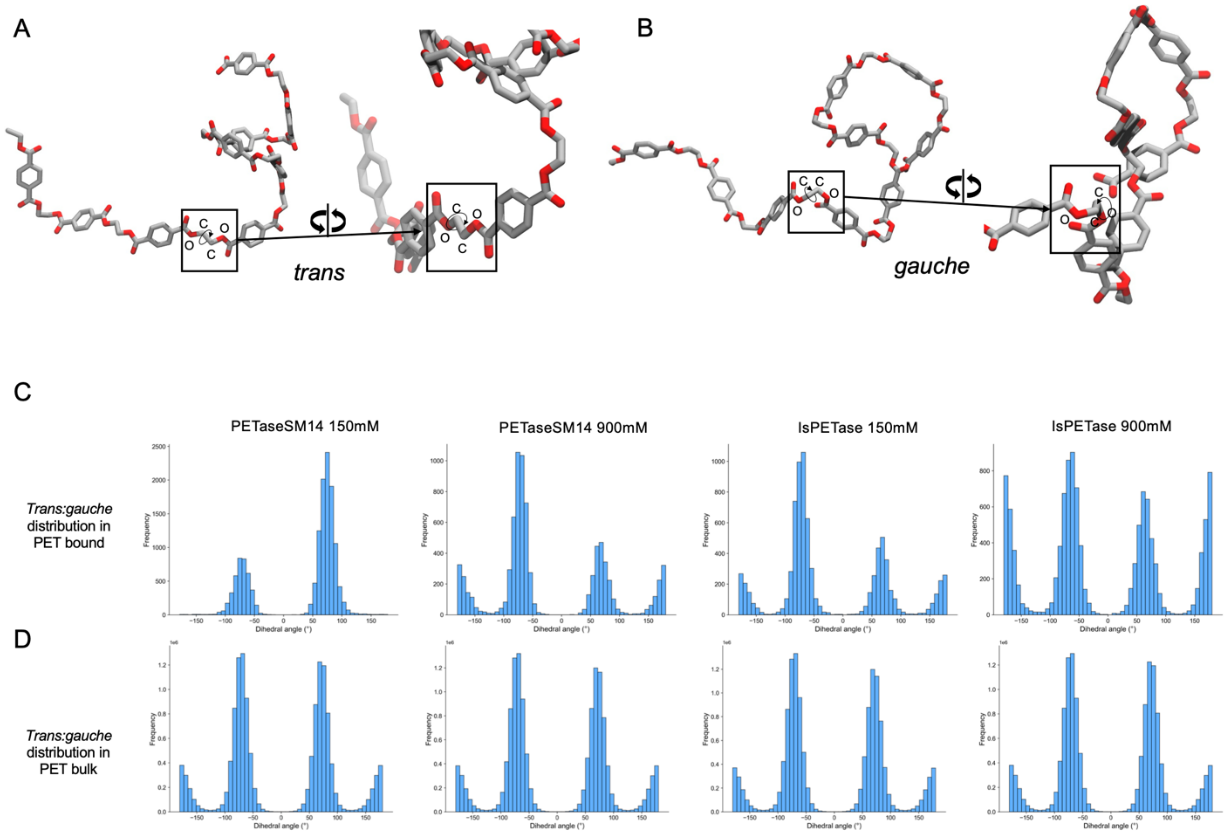

Figure S5. *Trans:gauche* conformational distribution of PET chains. Representative structures of the dihedral angles defined by the O3 - C9 - C10 - O1 atoms of PET chain in the (**A**) *trans* and (**B**) *gauche* conformation. *Trans:gauche* distribution of the PET chains (**C**) bound to the enzymes' binding sites or (**D**) in the PET bulk, averaged over the three 500-ns MD simulation replicas.

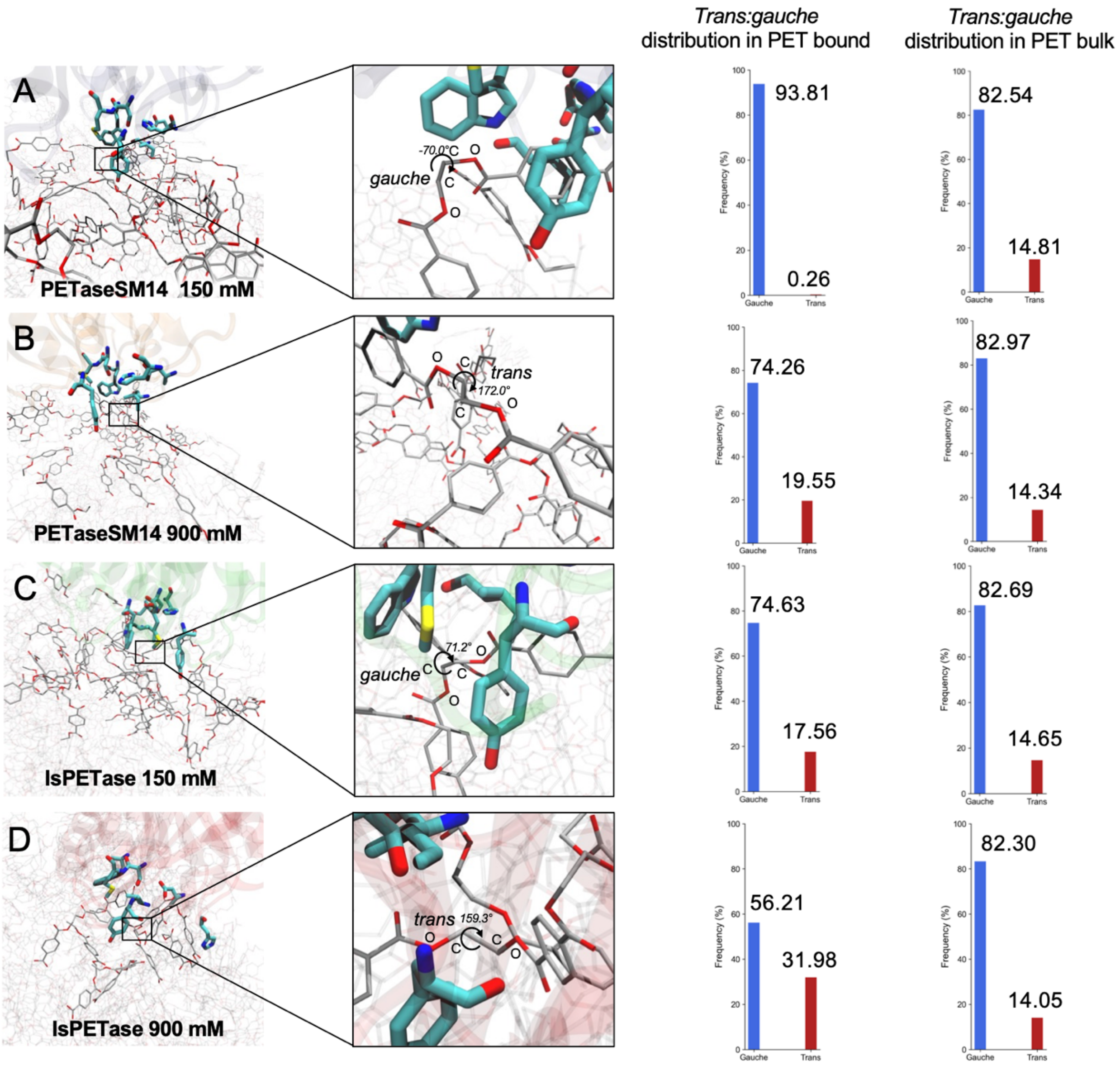

Figure S6. Conformational distribution of PET chains. Representative snapshots (left) and *trans:gauche* distributions of the PET chains (right) bound to the enzyme binding site and in the substrate bulk for (**A**) PETaseSM14 at 150 mM, (**B**) PETaseSM14 at 900 mM, (**C**) IsPETase at 150 mM, and (**D**) IsPETase at 900 mM. For the PET bound to the substrate, the bar charts (left) report the dihedral angle distributions calculated for the fraction at < 8 Å than the catalytic serine, averaged over the three 500-ns MD simulation replicas. In the case of the PET in the substrate bulk, the bar charts (right) report the dihedral angle distributions calculated for the 100 9-mer PET chains of each system, excluding the fraction bound to the binding site, averaged over the three 500-ns MD simulation replicas.

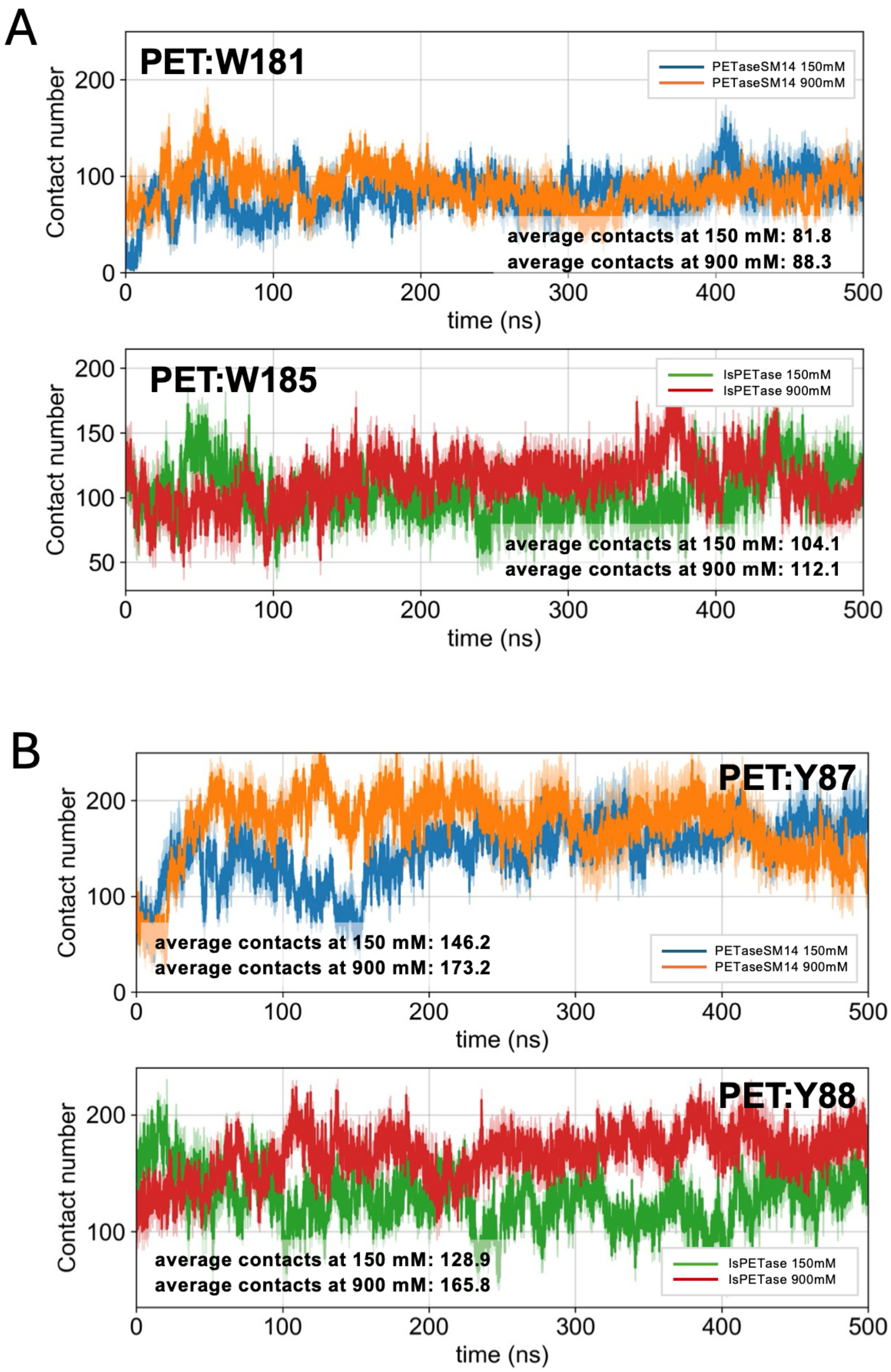

Figure S7. Contact number between the aromatic side chains within the active and the PET benzene groups. The time traces of the contacts number calculated between the side chains of either (**A**) the tryptophan (W181/W185) or (**B**) the tyrosine (Y88/Y87) and the PET benzene groups. The value and the associated error are reported as the average and standard deviation calculated over three independent replicas.

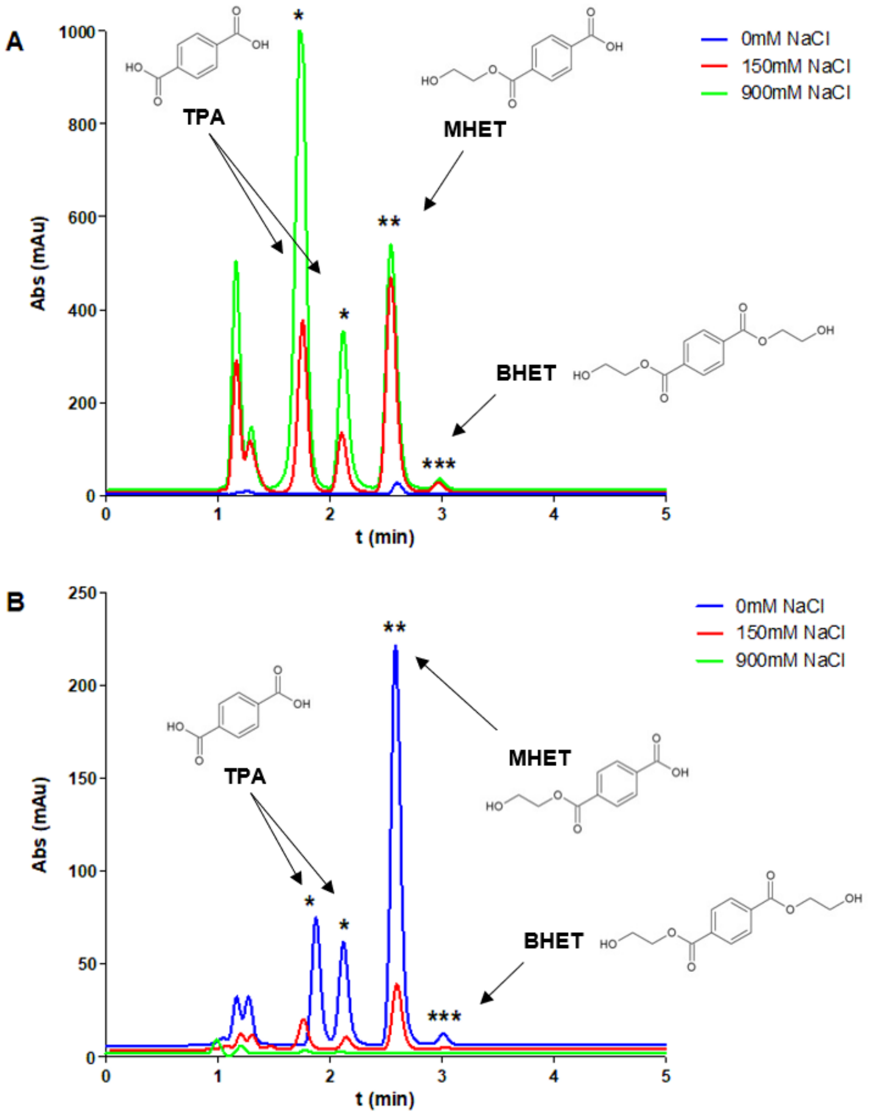

Figure S8. PETase reaction products released at different salt concentrations. HPLC chromatograms of the hydrolysis products present in the supernatant obtained after the digestion with PETaseSM14 (**A**) and IsPETase (**B**) on PET powder after 72 h of incubation at 37°C with 400 rpm at different NaCl concentration: 0 mM (blue line), 150 mM (red line), 900 mM (green line). The separation occurred as described in the Materials and Methods section. For the sake of clarity, only the region in which the reaction products are eluted is shown, and the peaks of interest have been marked at the top with stars: TPA* (1.7 and 2.1 min), MHET** (2.5 min), BHET*** (2.9 min). The lines colors, based on the salt concentration, remained consistent between the two graphs but the order was reversed for greater clarity; in fact, with PETaseSM14 (**A**) at the increase of salt concentration a significant increase of products concentration is observed while using IsPETase (**B**) at high salt concentrations (green line) the release of products is practically zero.

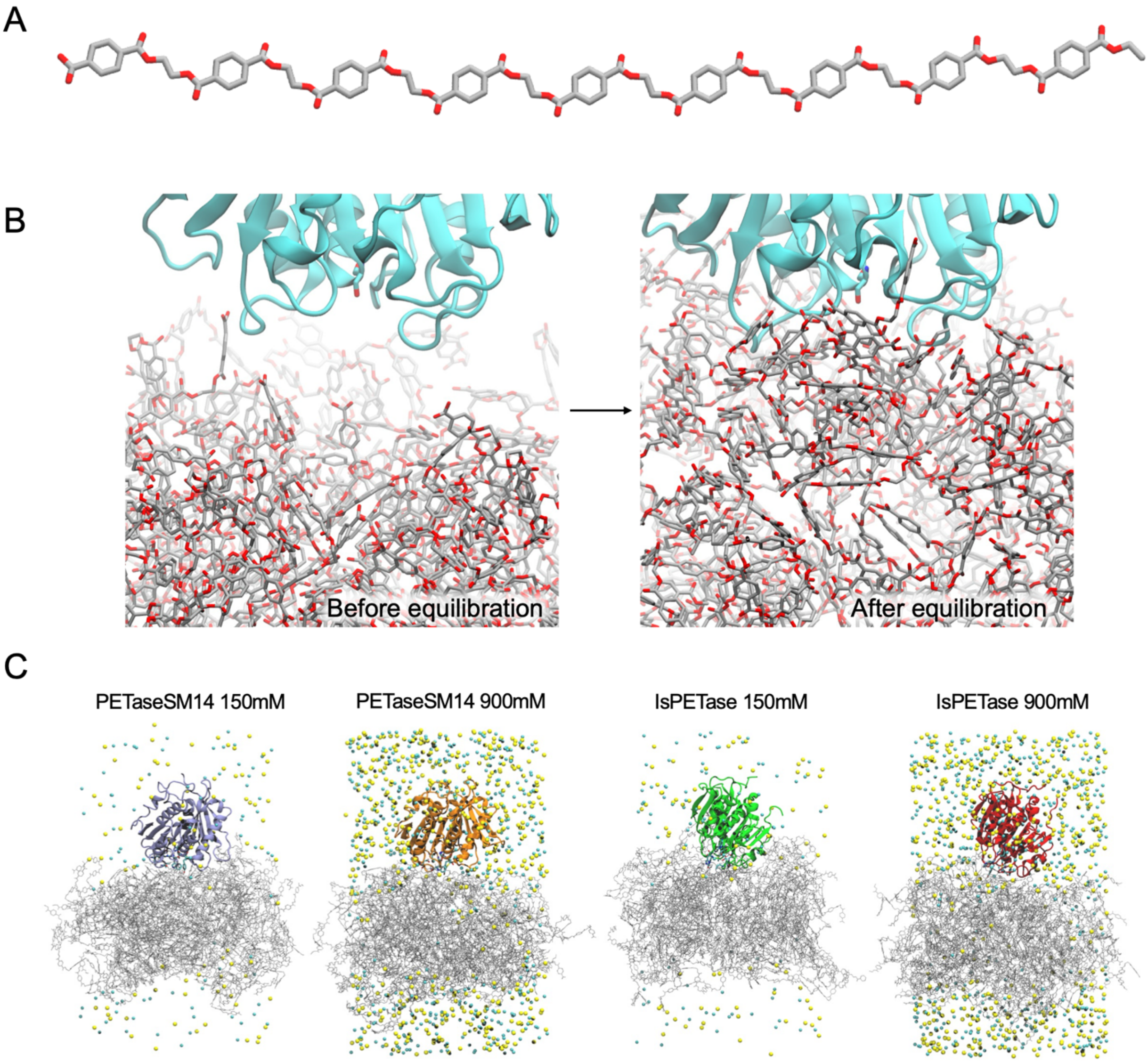

Figure S9. Enzymes adsorbed onto PET slabs simulation systems. (**A**) Single PET9 chain in linear conformation provided by CHARMM-GUI, before simulated annealing and equilibration. (**B**) Interface between the PETaseSM14 binding site (at 150 mM of NaCl concentration) and the PET slab surface before (left panel) and after (right panel) equilibration. (**C**) Starting configuration of PETaseSM14 and IsPETase simulation systems, adsorbed onto the PET slab and surrounded by the Na^+ (cyan spheres) and Cl^- (yellow spheres) at 150 mM and 900 mM of ion concentration. The proteins are shown with the cartoon representation using the same colors as those used in the analysis reported in this work. The PET chains are colored in grey, while water molecules are not shown for the sake of clarity.

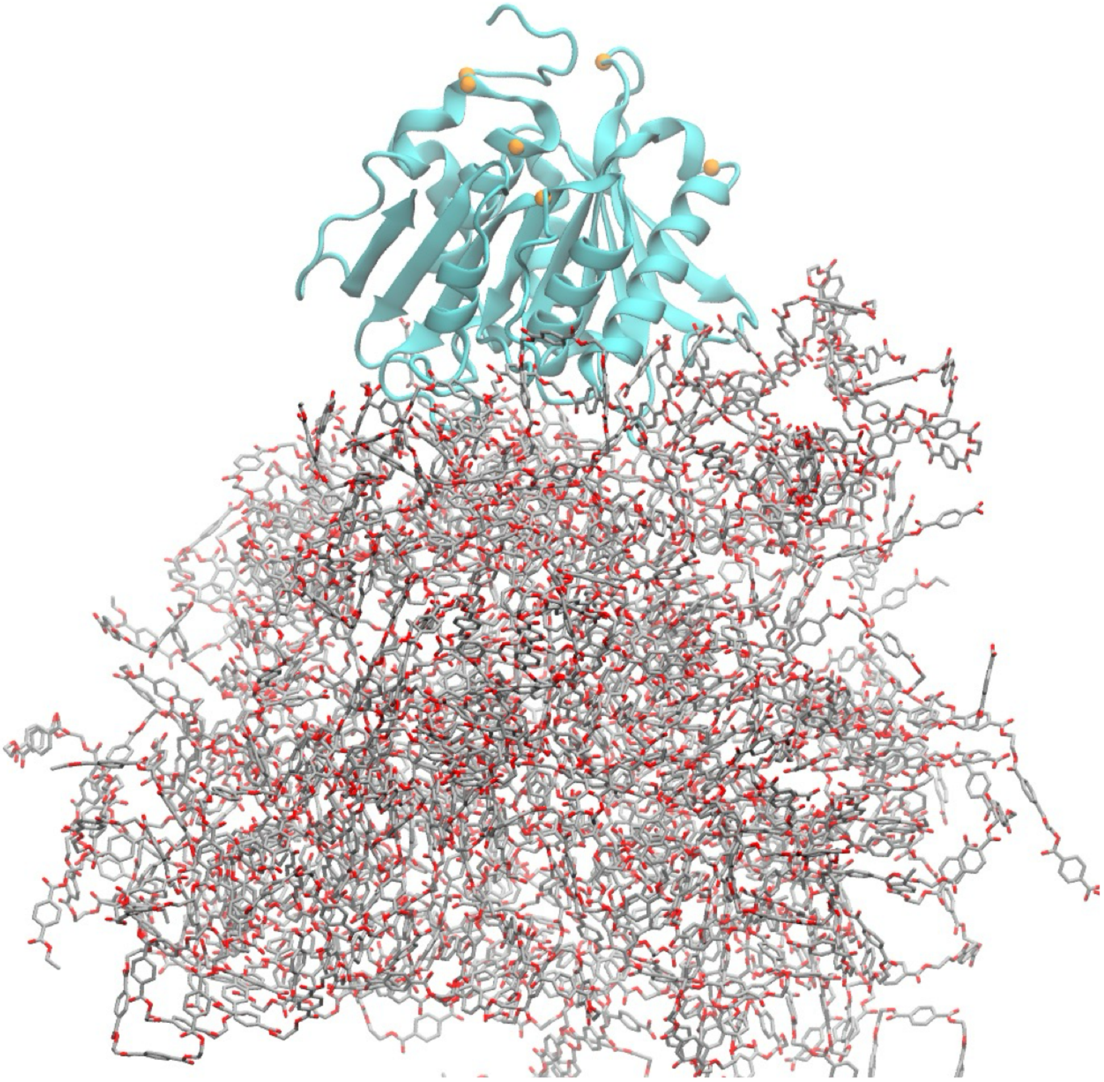

Figure S10. Positional restraints applied to the enzymes. The minimal restraints applied to few Cα atoms of the enzymes far from the binding sites are indicated with the orange Van der Waals spheres.

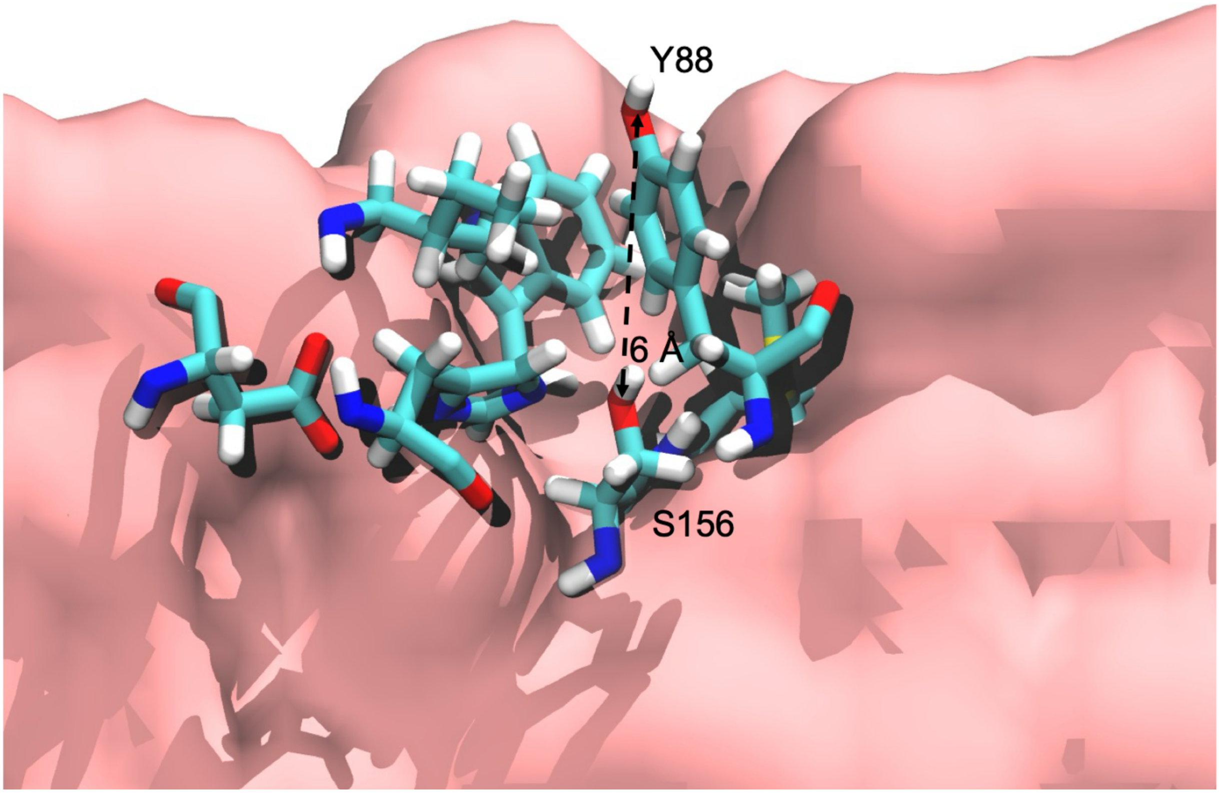

Figure S11. Definition of the cutoff for water molecules in the active site. [MCM1]The cutoff used to define the number of water molecules that entered the binding site was selected as the distance between the Oγ atom of the catalytic serine and the Oγ atom of the tyrosine (Y87/Y88). The PETaseSM14 binding site is shown.

Table S1. Solvation of the enzymes' binding site from molecular dynamics simulations. The solvent-accessible surface area (SASA) and the number of water molecules within the binding sites are reported as the average value over the three replicas.

	PETaseSM14		IsPETase	
	**150 mM**	**900 mM**	**150 mM**	**900 mM**
**SASA**	326.7 $Å^2$	356.7 $Å^2$	481.9 $Å^2$	479.9 $Å^2$
**Number of water molecules**	4.10	6.32	6.88	7.32

Table S2. Equilibration phase of the enzymes adsorbed onto the PET slab systems. The 50-ns equilibration protocol was divided into five sequential steps of 10 ns each. For every step, the time step, simulation duration, ensemble, and applied restraints are specified. Each of the three replicas per system was equilibrated independently following the same protocol.

Equilibration step	Time step	Simulation time	Ensemble	Restraints
STEP 1	1 fs	10 ns	NVT	Each atom except water
STEP 2	2 fs	10 ns	NVT	Heavy atoms of protein and PET
STEP 3	2 fs	10 ns	NVT	Heavy atoms of the protein
STEP 4	2 fs	10 ns	NPT	Cα atoms of the protein
STEP 5	2 fs	10 ns	NPT	Cα atoms of residues 35, 71, 76, 140, 192, 255, and 260 of the protein
